# Long-term sucralose exposure accelerates ovarian aging via gut microbiota dysbiosis

**DOI:** 10.1038/s41538-026-00775-x

**Published:** 2026-03-24

**Authors:** Junfeng Chen, Donghai Zhang, Jie Gao, Ziyi Zhang, Junjie Qu, Yiran Li

**Affiliations:** 1 Shanghai Key Laboratory of Maternal Fetal Medicine, Shanghai Institute of Maternal-Fetal Medicine and Gynecologic Oncology, Shanghai First Maternity and Infant Hospital, School of Medicine, Tongji University, Shanghai, China; 2 Center for Reproductive Medicine, Shanghai First Maternity and Infant Hospital, School of Medicine, Tongji University, Shanghai, China

**Keywords:** Diseases, Microbiology

## Abstract

The increasing prevalence of obesity, diabetes, and other metabolic disorders is driving the application of sucralose in the food industry. However, its direct effects on the reproductive function have not been sufficiently investigated. Chronic sucralose consumption may induce ovarian aging, leading to ovarian dysfunction. This occurs in association with intestinal dysbiosis and disruption of intestinal tight junctions caused by prolonged intake of sucralose. These changes increase the production of lipopolysaccharide (LPS), which is derived from gram-negative bacteria, and facilitate its translocation into the systemic circulation. Circulating LPS binds to TLR4 in ovarian granulosa cells, activating the NF-κB signaling pathway. This activation triggers the nuclear translocation of the p65 subunit and increases the transcription of downstream inflammatory cytokines. Moreover, Baicalin methyl ester may serve as an early intervention strategy. It can significantly attenuate both intestinal and ovarian inflammatory damage induced by sucralose, primarily by inhibiting the TLR4/NF-κB pathway.

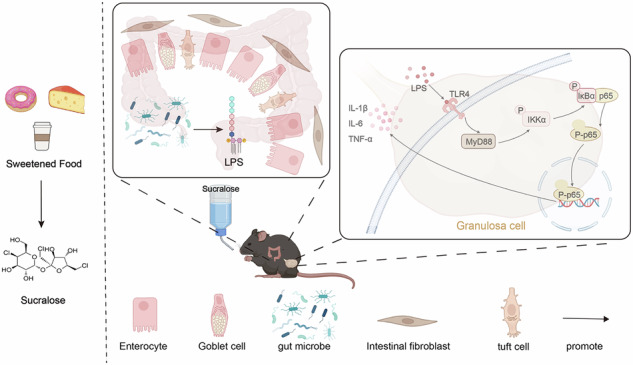

## Introduction

Artificial sweeteners have been used in various drugs, foods, drinks, and hygiene products in the last few decades^1^. Sucralose, a non-caloric sweetener, has about 600-fold greater sweetness than sucrose and is a frequently used artificial non-nutritive sweetener^2^. Sucralose absorption is restricted by its stable characteristics, with only 11–27% absorbed in the human body^2,3^. Sucralose can be detected in the bloodstream after the consumption of sucralose-containing foods or beverages^4^. The consumption of 250 mg of sucralose leads to plasma levels of about 1 μM within 90–120 min^4,5^. Some studies have shown that use of artificial sweeteners may be linked to various negative health effects, such as weight gain, depression, anxiety, imbalance in the gut flora, and risk of developing cancer^6–9^. The effect of artificial sweeteners on human reproductive health remains mostly unknown.

Ovaries play an important role in the fertility of females^10^. Ovaries age earlier in the human body, and their function declines significantly earlier (after the age of 35) than most internal organs (including the liver, heart, or kidneys), which generally decline in function after the age of 50–60 years^11–13^. Ovarian aging is characterized by a reduction in follicle quantity and quality, manifested as menstrual cycle disorders or even amenorrhea, a gradual decline in fertility to complete loss of fertility, and the occurrence and development of various systemic diseases, ultimately progressing to menopause^14–16^. When the body is in a chronic inflammatory environment for a long period, the overexpression of inflammatory factors, including interleukin-6 (IL-6) and tumor necrosis factor alpha (TNF-α), can lead to a sustained, highly pro-inflammatory state, inducing irreversible aging-related changes in ovarian tissue^13^. TNF-α, IL-6, and other factors are associated with decreased ovarian reserve^13,17^. As individuals age, the serum levels of the abovementioned inflammatory factors in aging organs increase significantly, especially in the ovaries of mice with ovarian aging^18^. After treatment with anti-inflammatory compounds, damaged ovarian function is repaired^19^. This suggests that the occurrence of ovarian aging may be prevented through anti-inflammatory measures^17–19^.

Baicalin is a flavonoid compound isolated from the dried roots of the plant *Scutellaria baicalensis* Georgi^20^. It has broad-spectrum antibacterial effects, as well as anti-inflammatory, anticancer, antivirus, and anti-allergic activities^20^. Baicalin, a flavonoid glycoside, is an effective inhibitor of the TLR4/NF-κB pathway^21^. It blocks inflammatory responses through multiple mechanisms, such as targeting TLR4 signaling, stabilizing IκBα, inhibiting NF-κB nuclear transport, and upregulating the NF-κB inhibitor IκB protein^22^. In the DSS-induced colitis model, baicalin indirectly protects the ovarian microenvironment by repairing intestinal barrier function and reducing the leakage of blood lipopolysaccharide (LPS)^23,24^. Owing to the low absorption efficiency of baicalin, increasing its bioavailability through chemical modification or formulation improvement is necessary to increase its therapeutic efficacy by targeting key pathways^25^. Therefore, baicalin methyl ester (BME), a “novel” compound that esterifies hydroxyl-containing drugs, has emerged. BME improves its water solubility through chemical modification, thereby increasing its absorption efficiency after oral administration^24,25^. This can solve the problem of the poor absorption efficiency of traditional baicalin and improve the controllability of its therapeutic effects. In addition, BME are used as therapeutic drugs based on their dual and synergistic pharmacological mechanisms. Studies have shown that BME not only inhibits the biosynthesis of LPS in Gram-negative bacteria^21^, but also directly antagonizes the TLR4/NF-κB inflammatory signaling pathway^22^. This is highly consistent with the core pathological feature of “LPS accumulation TLR4/NF-κB overactivation” in the disease model used in this study, aimed at synchronously intervening in the triggering and amplification of inflammation^23,25^. Therefore, regulating inflammation and oxidative stress may be key strategies to combat ovarian aging.

Our findings suggested that long-term sucralose intake causes chronic enteritis in mice, manifested as damage to the intestinal barrier, a significant increase in blood LPS levels, and activation of the TLR4/NF-κB pathway in ovarian granulosa cells, consistent with an inflammation-linked mechanism contributing to ovarian aging. Further studies revealed that baicalin intervention can effectively block this process by restoring intestinal tight junction protein levels, decreasing blood LPS levels, and directly inhibiting the activation of the TLR4/NF-κB pathway in granulosa cells. In this study, we elucidated the mechanism by which sucralose accelerates ovarian aging through gut ovarian cross-organ interactions and confirmed that baicalin reverses this pathological process through multitarget interventions such as intestinal barrier repair and TLR4/NF-κB inhibition. This study provided a new intervention strategy for reproductive health risks associated with dietary additives.

## Results

### Long-term intake of sucralose causes aging in multiple organs

**Fig. 1:**
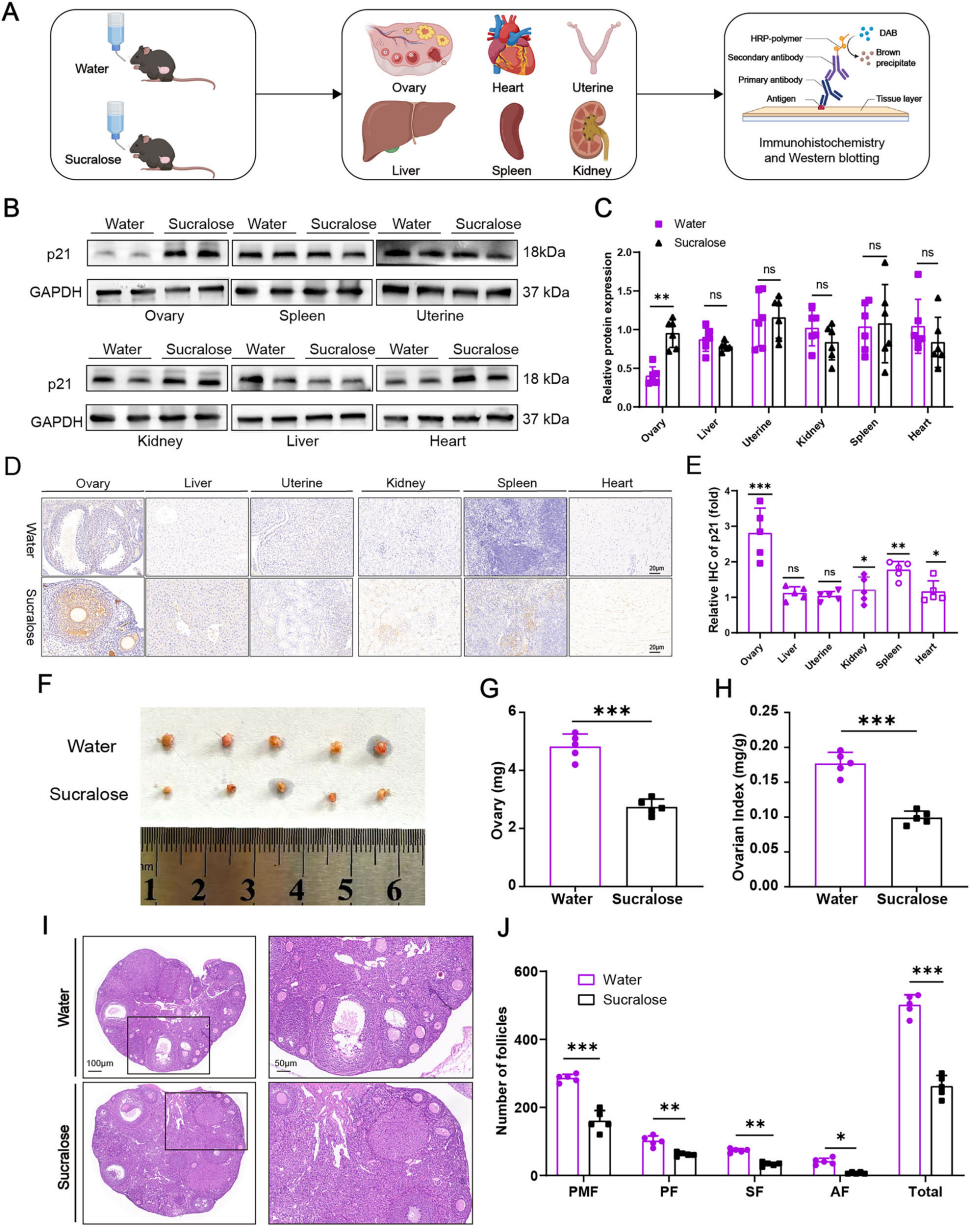
Long-term intake of sucralose causes aging in multiple organs. **A** Schematic representation of the experimental design. **B** Western blot analysis of the senescence marker p21 in ovaries and other organs from water and long-term sucralose-treated mice. **C** Quantification of p21 protein levels normalized to GAPDH in multiple tissues (*n* = 6 mice per group). **D** Representative IHC staining of p21 in ovaries and other organs from water and long-term sucralose-treated mice. Scale bars = 20 μm. **E** Quantification of the relative expression of IHC staining in ovaries and other organs (*n* = 5 mice per group). **F** Representative images of mice ovaries from two groups. **G**, **H** Ovary weight and ovary index of water and long-term sucralose-treated mice (*n* = 5 mice per group). **I** Representative H&E staining ovarian sections from water and long-term sucralose-treated mice. Scale bars = 100 μm (left) and 50 μm (right). **J** Numbers of ovarian follicles at different stages, including the primordial follicle (PMF), the primary follicle (PF), the secondary follicle (SF) and the antral follicle (AF) (*n* = 5 mice per group). The data represent the mean ± SEM. *p < 0.05, **p < 0.01, and ***p < 0.001.

Sucralose (0.1 mg/mL) was added to the drinking water of the mice. After short-term treatment for 2 weeks and long-term treatment for 3 months, samples of various organ tissues, including the ovary, heart, uterus, liver, kidney, and spleen, were collected. Tissue samples were collected from various organs for WB and IHC analyses to compare the expression of the senescence marker p21 in the water and sucralose groups (Fig. 1A). The results of the IHC and WB assays suggested that the expression of the senescence marker p21 did not increase considerably in various organs of the mice in the short-term treatment group (Fig. S1B, C). When comparing the tissues of mice that have been taking sucralose for a long time, IHC and WB assays revealed significantly upregulated expression of the senescence marker p21 in the ovaries of the mice in the long-term treatment group. This phenomenon has been found in other organs, but not as significant (Fig. 1B–E). These results indicate that long-term sucralose intake in mice causes aging of organs and that ovarian aging occurs earlier than in most other organs.

### Long-term intake of sucralose accelerates ovarian aging and functional impairment

**Fig. 2:**
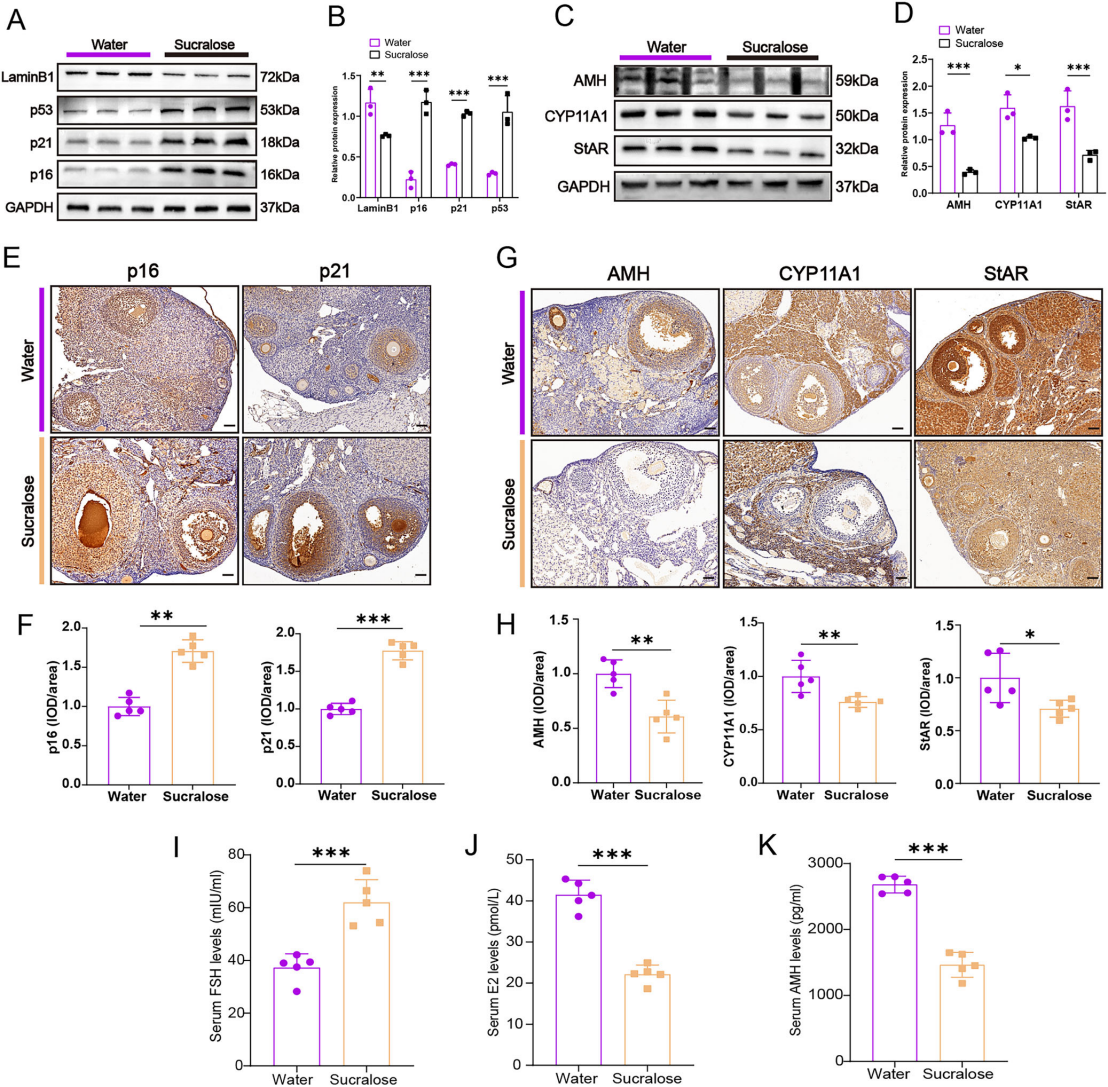
Long-term intake of sucralose accelerates ovarian aging and functional impairment. **A**, **B** Western blot bands and relative protein expressions of Lamin B1, p53, p21, and p16 in ovaries tissues (*n* = 3 mice per group). **C**, **D** Western blot bands and relative protein expressions of AMH, CYP11A1, StAR in ovaries tissues (*n* = 3 mice per group). **E**, **F** IHC-staining images and analyses of Lamin B1, p53, p21, and p16 proteins in ovarian tissues (*n* = 5 mice per group). Scale bars = 50 μm. **G**, **H** IHC-staining images and analyses of AMH, CYP11A1, StAR proteins in ovarian tissues (*n* = 5 mice per group). Scale bars = 50 μm. **I**–**K** Serum concentrations of FSH, E2, and AMH (*n* = 5 mice per group). The data represent the mean ± SEM. *p < 0.05, **p < 0.01, and ***p < 0.001.

During 3 months of treatment, body weight was recorded once a week (Fig. S1A). While the body weights of the mice showed no significant difference between the two groups, mice subjected to long-term sucralose presented a significantly lower ovarian tissue weight and a significantly lower ovarian index (ovarian weight/body weight) than the mice in the control group (Fig. 1F–H). The ovarian reserve is generally assessed by quantifying follicles at various developmental stages^11,26^. To investigate potential morphological alterations in the ovaries of mice chronically exposed to sucralose, we performed H&E staining on ovarian slices and counted the follicles. The analysis revealed a significant reduction in the number of antral follicles in the sucralose-treated mice compared to that in the control mice (Fig. 1I, J). Additionally, the number of primordial, primary, secondary, and the total follicle count were significantly lower in the sucralose-treated ovaries (Fig. 1I, J). However, estrous cycles showed the water group displayed more stable and consistent cycles, whereas the sucralose group showed minor fluctuations in stage duration without overt disruption of cycle rhythm (Fig. S1D). We conducted WB analysis to further detect aging-related indicators in ovarian tissue^27^. The levels of the senescence markers p53, p21, and p16 markedly increased in the ovarian tissues of the sucralose group when compared with those of the control group, whereas the LaminB1 levels were significantly reduced (Fig. 2A, B). CYP11A1 and StAR are key proteins reflecting estrogen synthesis, whereas AMH is a critical regulator of follicular development^28,29^. The expression levels of CYP11A1, StAR, and AMH in ovarian tissues from sucralose-treated versus control mice are shown in Fig. 2C, D. The results of the IHC analysis revealed greater expression of ovarian senescence markers, particularly p16 and p21, in granulosa cells in the sucralose group (Fig. 2E, F). Concurrently, the expression levels of STAR, CYP11A1, and AMH were reduced (Fig. 2G, H). Aligning with this finding, the serum AMH and E2 levels markedly reduced in the sucralose groups relative to their levels in the control groups, whereas the FSH levels were significantly elevated (Fig. 2I–K). These results demonstrate that long-term consumption of sucralose accelerates ovarian aging and impairs ovarian function.

### Molecular mechanism of long-term intake of sucralose-induced ovarian aging

**Fig. 3:**
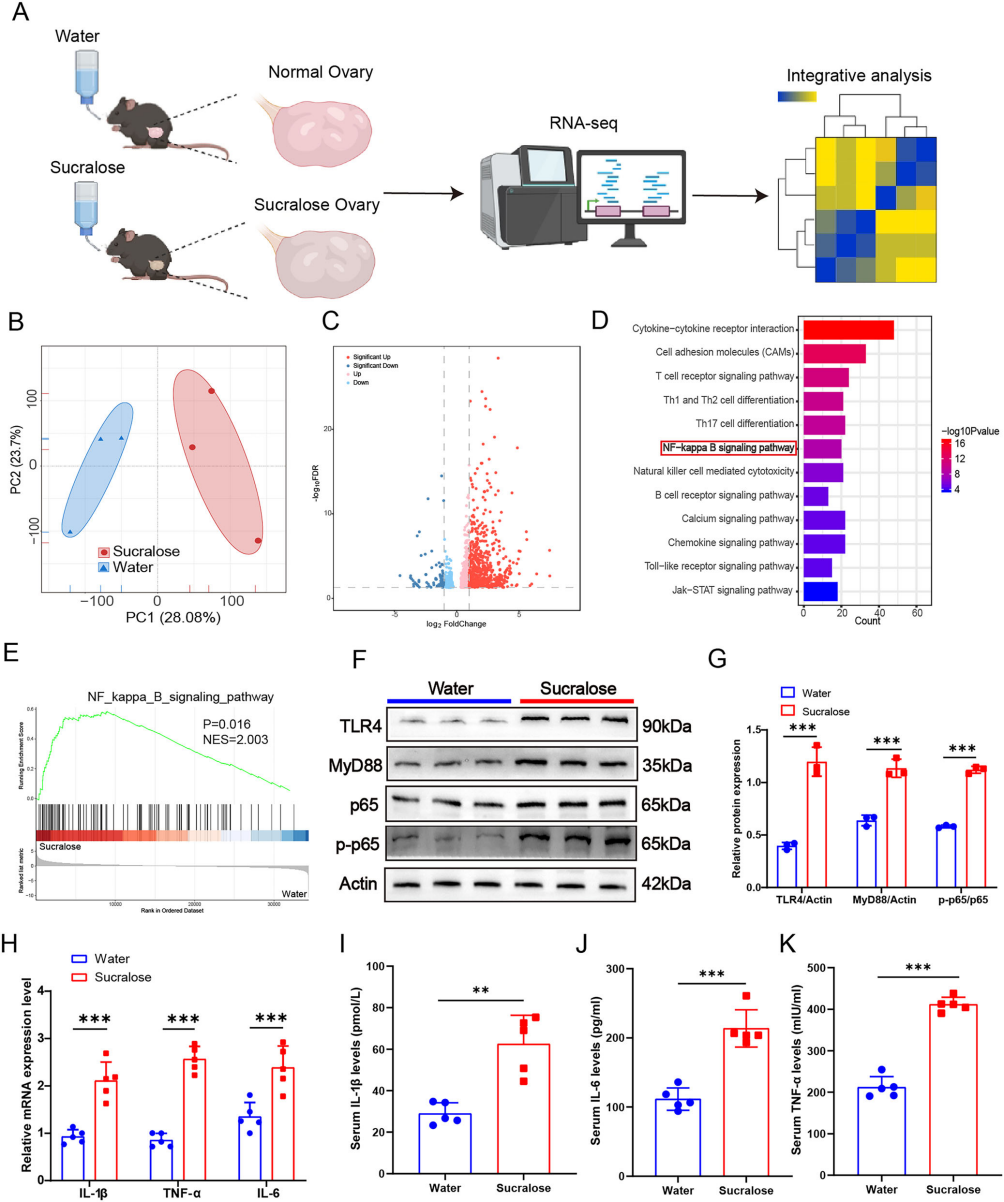
Molecular mechanism of long-term intake of sucralose-induced ovarian aging. **A** Workflow of ovarian tissue RNA-sequencing analysis. **B** Principal component analysis (PCA) was performed on the ovarian transcriptome data. **C** Volcano plot of differentially expressed genes identified from transcriptomic analysis of ovarian samples. **D** Scatter plots for upregulated pathways from the KEGG pathway analysis. **E** GSEA revealed enrichment of the NF-κB signaling pathway in the ovaries of mice subjected to long-term sucralose treatment. **F** Western blot images showing protein expression levels of TLR4, MyD88, and p-p65 in ovarian tissues (*n* = 3 mice per group). **G** Relative protein expression levels were quantified by normalizing band intensities to Actin or p65. **H** RT-qPCR analysis of relative mRNA expression of inflammatory cytokines (IL-1β, TNF-α, and IL-6). **I**–**K** Serum concentrations of inflammatory cytokines (IL-1β, TNF-α, and IL-6). The data represent the mean ± SEM. **p < 0.01, and ***p < 0.001.

To elucidate the mechanism related to the long-term intake of sucralose-induced acceleration of ovarian aging, we performed transcriptomic profiling of ovarian tissues from mice in the control and sucralose groups (Fig. 3A). Principal component analysis (PCA) of the RNA-seq data indicated that both groups were separated (Fig. 3B). Through differential analysis, we found that 1140 genes were upregulated, whereas 265 genes were downregulated in the sucralose group relative to their expression levels in the control group (Fig. 3C). According to the Kyoto Encyclopedia of Genes and Genomes (KEGG) analysis, most upregulated genes were related to the NF-κB pathway in the sucralose group (Fig. 3D). Additionally, GSEA revealed significant enrichment of the activated NF-κB pathway in ovaries in the sucralose group (Fig. 3E). These findings indicate that long-term exposure to sucralose activates the NF-κB pathway in ovaries. WB analysis confirmed significantly increased TLR4, Myd88, p-p65/p65, and IKKα/p-IKKα levels in ovarian tissues of the sucralose-treated mice (Figs. 3F, G and S2A, B). To assess the expression of inflammatory cytokines downstream of the NF-κB pathway, RT-qPCR analysis was carried out, and the results revealed elevated IL-1β, IL-6, and TNF-α expression in the ovarian tissues of the sucralose-treated mice (Fig. 3H). Moreover, the ELISA results also confirmed their increased levels (IL-1β, IL-6, and TNF-α) in the serum (Fig. 3I–K).

### Long-term intake of sucralose impairs intestinal barrier integrity and induces gut microbial dysbiosis

**Fig. 4:**
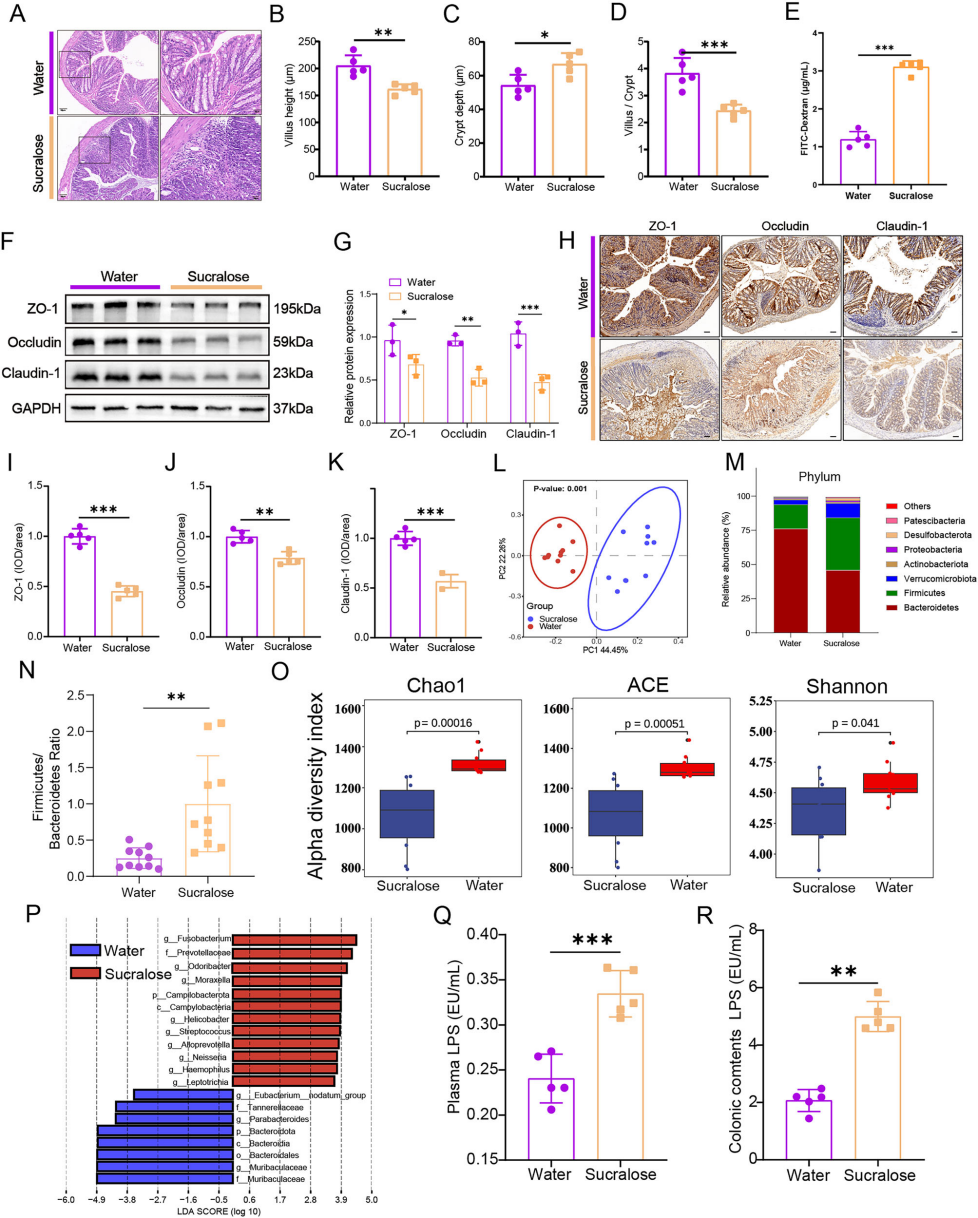
Long-term intake of sucralose impairs intestinal barrier integrity and induces gut microbial dysbiosis. **A** Representative H&E staining images of intestinal sections in mice. Scale bars = 100 μm (left) and 50 μm (right). **B**–**D** Comparison of villi height, crypt depth, and villi height/crypt depth in intestine samples of mice (*n* = 5 mice per group). **E** The plasmatic concentration of FITC-dextran. **F**, **G** Western blot bands and relative protein expressions of ZO-1, Occludin, and Claudin-1 in intestinal tissues. **H**–**K** IHC-staining images and analyses of ZO-1, Occludin, and Claudin-1 proteins in intestinal tissues (*n* = 5 mice per group). Scale bars = 50 μm. **L** Principal coordinates analysis (PCoA) of beta diversity representing gut microbiota composition in water and long-term sucralose-treated mice. **M** Phylum-level composition of bacterial communities in the indicated groups. **N** The ratio of Firmicutes to Bacteroidetes was determined based on 16S rRNA gene sequencing data. **O** Alpha diversity and richness of the gut microbiota, evaluated via the Chao1, ACE, and Shannon indices, were compared between water and sucralose group mice. **P** LEfSe analysis identified gut microbiota bacterial genera exhibiting significantly different abundances between water and sucralose-treated mice, as indicated by LDA scores (log10). **Q**, **R** Concentration of LPS in plasma and colonic contents (*n* = 5 mice per group). The data represent the mean ± SEM. *p < 0.05, **p < 0.01, and ***p < 0.001.

To evaluate how sucralose affects the intestinal barrier, we performed histopathological analysis via H&E staining and assessed the expression of intestinal tight junction proteins in mouse colon tissue. In the normal group, the colonic mucosa appeared intact and smooth, with preserved tissue architecture and no evidence of inflammatory cell infiltration (Fig. 4A). However, H&E staining revealed glandular architectural disruption, a reduction in the number of goblet cells, crypt damage, and extensive inflammatory cell infiltration in the sucralose group (Fig. 4A). Further H&E analysis indicated that the villus height was obviously lower and the crypt depth was significantly greater in the sucralose group, resulting in a significantly reduced villus-to-crypt ratio (Fig. 4B–D). Moreover, the sucralose group presented a significant reduction in colon length (Fig. S3A, B). These observations strongly indicate that sucralose has a significant damaging effect on the structure of colonic tissue. To evaluate the integrity of the intestinal barrier in mice, the study used fluorescein isothiocyanate labeled dextran for detection. As a result, it was found that compared with the control group that only drank water, mice that ingested sucralose had significantly increased levels of FITC glucan in their bodies (Fig. 4E). Moreover, colon tissue samples from both groups were collected to evaluate the levels of tight junction proteins (claudin-1, occludin, and ZO-1). The results of the WB analysis (Fig. 4F, G) revealed markedly reduced levels in sucralose-treated mice, and IHC (Fig. 4H–K) also confirmed considerably lower tight junction protein levels in the tissue sections. Although sucralose is almost not absorbed by the body, it may indirectly affect health by altering the gut microbial composition and activities^30,31^. We subsequently performed high-throughput 16S rRNA gene sequencing to characterize the taxonomic characteristics of the gut microbiota in each group of mice. Unweighted principal coordinate analysis (PCoA) revealed significantly different bacterial compositions between the two groups (Fig. 4L). At the phylum level, the mice in the sucralose group presented higher Firmicutes but lower Bacteroidetes abundances (Figs. 4M and S3C, F), as well as a significantly higher Firmicutes-to-Bacteroidetes (F/B) ratio (Fig. 4N). At the genus level, the abundances of the main genera significantly differed between the two groups. Relative to the mice in the water group, the mice in the sucralose group presented dysbiosis of the gut microbiota, manifested by a low abundance of *Muribaculaceae* and *Akkermansia* (Fig. S3E). Additionally, α diversity indices, such as the Chao1, ACE, Shannon (Fig. 4O), and Richness (Fig. S4D) indices, confirmed that microbial species significantly differed between the water and sucralose groups. LEfSe analysis revealed significantly differentially abundant taxa between the water and sucralose groups (LDA score >2). The sucralose group was enriched with LPS-producing and pro-inflammatory genera such as *Fusobacterium*, *Prevotella*, *Campylobacter*, and *Haemophilus*, suggesting a potential association with gut inflammation and increased LPS levels (Fig. 4P). In contrast, the water group was enriched in *Muribaculaceae, Parabacteroides*, and *Eubacterium nodatum*, which are associated with enhanced intestinal barrier function and reduced inflammatory potential (Figs. 4P and S3G). Therefore, we first found through experiments that colonic LPS levels in the sucralose group were elevated (Fig. 4Q). However, an increase in the LPS level in the colon contents does not necessarily indicate an increase in the LPS level in the bloodstream. Therefore, we detected the LPS level in mouse plasma and found that the plasma LPS levels in the sucralose group were higher relative to those in the control group (Fig. 4R).

### FMT in healthy mice reproduced the effect induced by long-term intake of sucralose

**Fig. 5:**
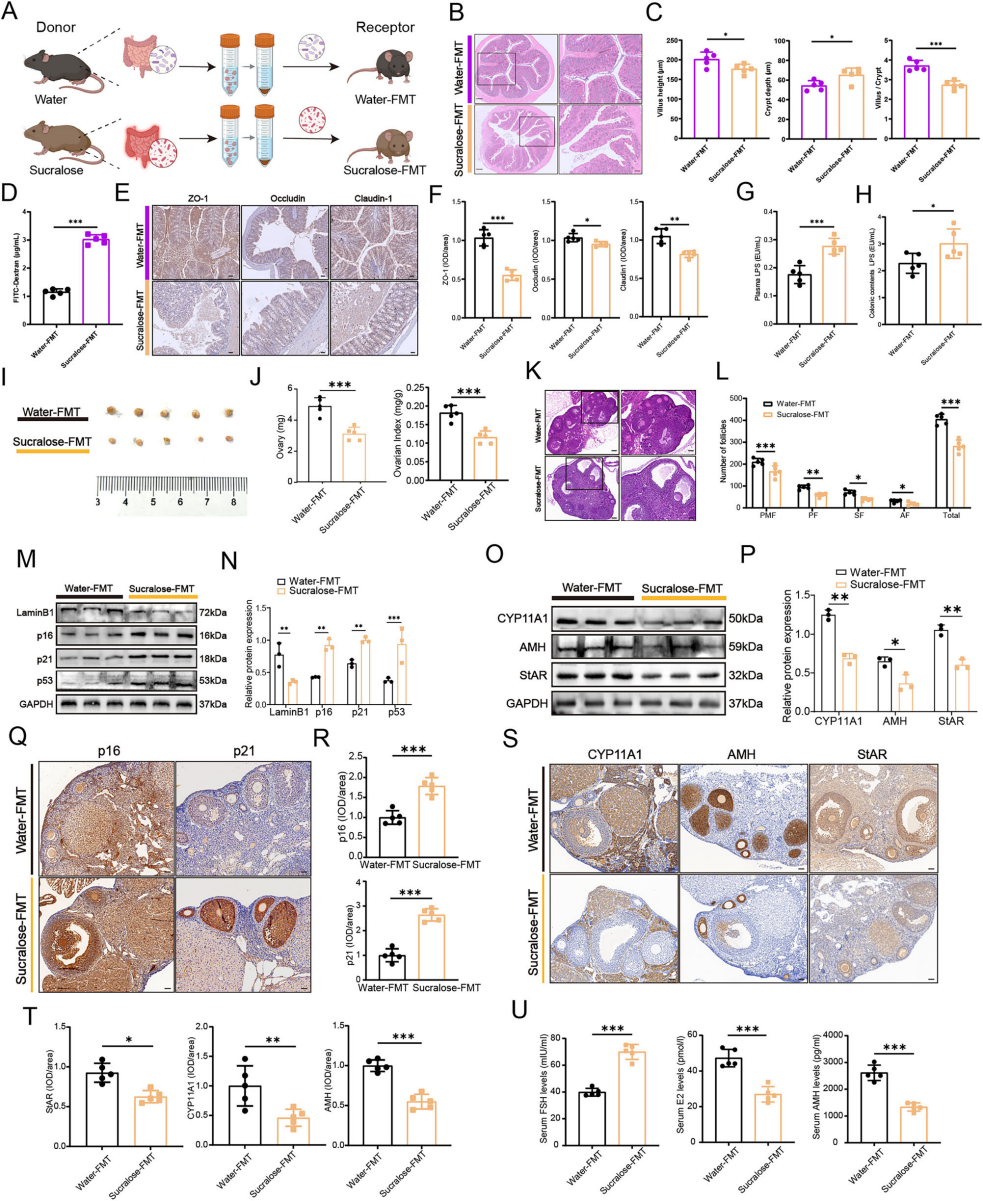
FMT in healthy mice reproduced the effect induced by long-term intake of sucralose. **A** Schematic diagram illustrating the procedure of the FMT experiment. **B** Representative H&E staining images of intestinal sections following FMT experiment Scale bars = 100 μm (left) and 50 μm (right). **C** Comparison of villi height, crypt depth, and villi height/crypt depth in intestine samples of mice following FMT experiment (*n* = 5 mice per group). **D** The plasmatic concentration of FITC-dextran in water-FMT and sucralose-FMT mice. **E**, **F** IHC-staining images and analyses of ZO-1, Occludin, and Claudin-1 proteins in intestinal tissues in water-FMT and sucralose-FMT mice (*n* = 5 mice per group). Scale bars = 50 μm. **G**, **H** Concentration of LPS in plasma and colonic contents in water-FMT and sucralose-FMT mice (*n* = 5 mice per group). **I** Representative images of mouse ovaries following FMT experiment (*n* = 5 mice per group). **J** Ovary weight and ovary index of water-FMT and sucralose-FMT mice (*n* = 5 mice per group). **K** Representative H&E staining ovarian sections from water-FMT and sucralose-FMT mice. Scale bars = 100 μm (left) and 50 μm (right). **L** Numbers of ovarian follicles at different stages in water-FMT and sucralose-FMT mice (*n* = 5 mice per group). **M**, **N** Western blot bands and relative protein expressions of Lamin B1, p53, p21, and p16 in ovaries tissues of water-FMT and sucralose-FMT mice (*n* = 3 mice per group). **O**, **P** Western blot bands and relative protein expressions of AMH, CYP11A1, StAR in ovaries tissues of water-FMT and sucralose-FMT mice (*n* = 3 mice per group). **Q**, **R** IHC-staining images and analyses of Lamin B1, p53, p21, and p16 proteins in ovaries tissues of water-FMT and sucralose-FMT mice (*n* = 5 mice per group). Scale bars = 50 μm. **S**, **T** IHC-staining images and analyses of AMH, CYP11A1, StAR proteins in ovaries tissues of water-FMT and sucralose-FMT mice (*n* = 5 mice per group). Scale bars = 50 μm. **U** Serum concentrations of FSH, E2, and AMH in water-FMT and sucralose-FMT mice (*n* = 5 mice per group). The data represent the mean ± SEM. *p < 0.05, **p < 0.01, and ***p < 0.001.

We conducted FMT to directly alter the gut microbiota in recipient mice. To explore whether changes in the gut microbiota are sufficient to transmit ovarian aging-related phenotypes, we transplanted fecal microbiota from mice in the water group and the sucralose group into normal mice via oral gavage (Fig. 5A). The H&E staining results showed that compared with the water-FMT group, the sucralose-FMT group also exhibited significant pathological changes in intestinal tissue, including glandular structural disorder, reduced number of goblet cells, crypt structural damage, and extensive infiltration of inflammatory cells (Fig. 5B). Further morphometric analysis showed a significant decrease in villus height and an increase in crypt depth in the sucralose-FMT group, resulting in a significant decrease in the ratio of villus height to crypt depth (Fig. 5C). Compared to the water-FMT group, sucralose-FMT group showed a significant increase in FITC glucan levels in their bodies (Fig. 5D). The IHC results showed that compared with the water-FMT group, the levels of tight junction proteins (claudin-1, occludin, and ZO-1) in tissue sections of sucralose-FMT group were significantly reduced (Fig. 5E, F). Meanwhile, the levels of LPS in the colon contents and plasma of sucralose-FMT mice were higher than those in the water-FMT group (Fig. 5G, H). We found a significant reduction in ovary weight and the ovarian index in the sucralose-FMT group compared to those in the water-FMT group (Fig. 5I, J). H&E staining revealed a decrease in the number of follicles at all levels in the sucralose-FMT group, indicating a reduction in the ovarian reserve in the sucralose-FMT group (Fig. 5K, L). Western blotting analysis (Fig. 5M, N) and IHC (Fig. 5Q, R) revealed that the levels of ovarian senescence markers (p53, p21, and p16) were considerably higher in sucralose-FMT mice, whereas the levels of Lamin B1 and follicle development-related markers (CYP11A1, StAR, and AMH) were significantly lower (Fig. 5O, P, S, T). Moreover, the serum levels of AMH and E2 in the sucralose-FMT group were obviously lower than those in the water-FMT group, and the serum levels of FSH were obviously higher than those in the water-FMT group (Fig. 5U). While these findings indicate that gut microbiota alterations are sufficient to transmit intestinal and ovarian phenotypes, the specific microbial composition of post-FMT recipient mice was not directly characterized in this study.

### LPS induces the nuclear translocation of p65 and increases the transcription of downstream inflammatory factors

**Fig. 6:**
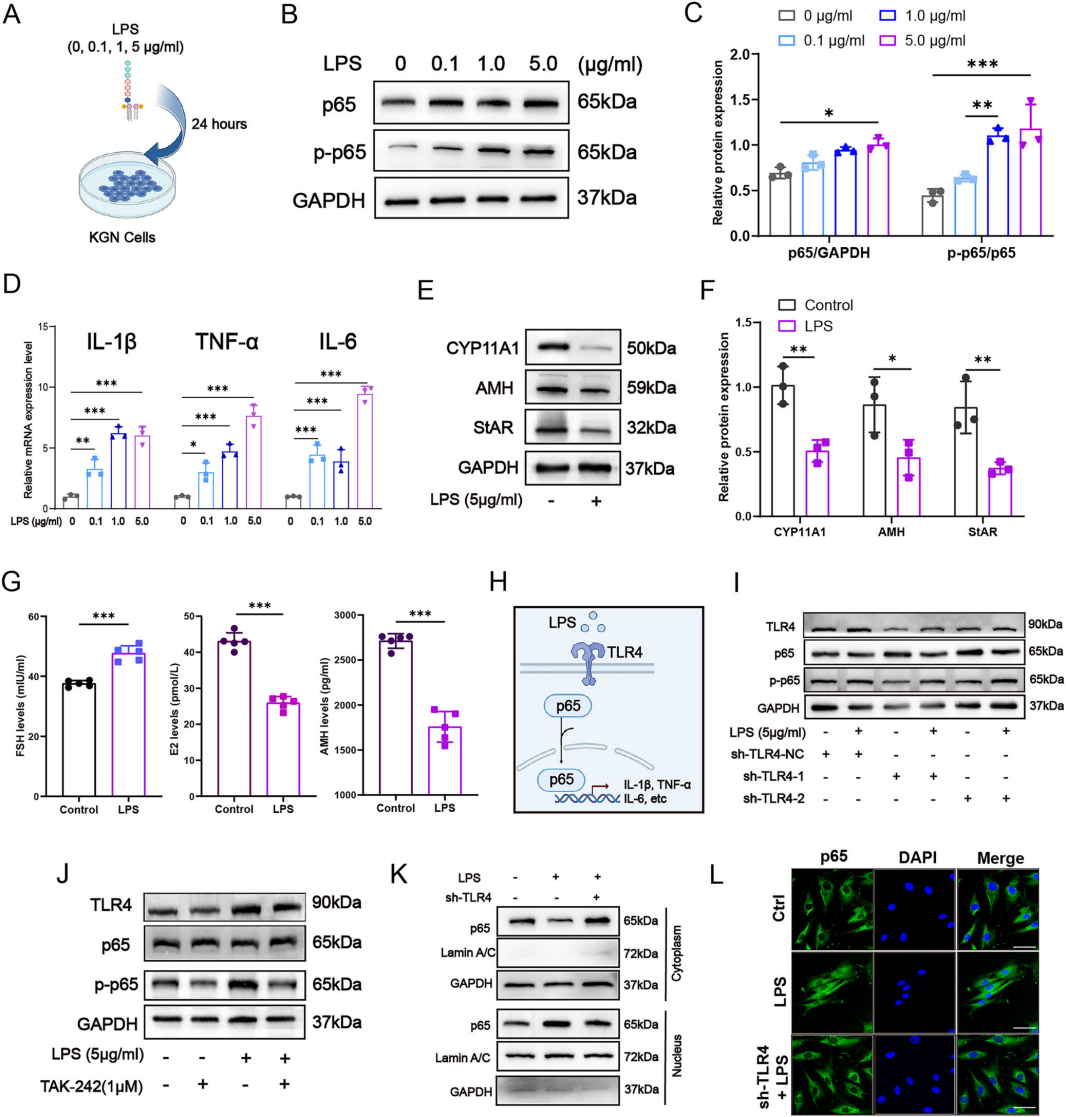
LPS induces the nuclear translocation of p65 and increases the transcription of downstream inflammatory factors. **A** Schematic illustration of LPS stimulation in KGN cells. **B**, **C** Western blot bands and relative protein expressions of p65 and phospho-p65 in KGN cells treated with different LPS concentrations (0, 0.1, 1.0, 5.0 μg/ml). **D** RT-qPCR analysis was performed to determine the relative mRNA expression of inflammatory cytokines (IL-1β, TNF-α, IL-6) in KGN cells treated with LPS at 0, 0.1, 1.0, and 5.0 µg/ml. **E**, **F** Western blots and relative protein expressions of CYP11A1, AMH and StAR in the control group and KGN cell LPS treatment group. **G** Concentrations of FSH, E2, and AMH in the cell culture supernatants of control and LPS-treated KGN cells. **H** Schematic illustration of the LPS signaling pathway mediated by TLR4. **I**, **J** Western blot bands of TLR4, p65 and phospho-p65 from different treated groups. **K** p65 level in the nuclear and cytoplasmic fractions was determined and normalized to Lamin A/C and GAPDH, respectively. **L** Immunofluorescence staining for p65. green: p65, blue: DAPI, scale bars = 50 μm. The data represent the mean ± SEM. *p < 0.05, **p < 0.01, and ***p < 0.001.

To determine the specific effects of LPS on ovarian granulosa cells, we added different concentrations of LPS (0, 0.1, 1, or 5 μg/mL) to KGN medium and cultured the cells for 24 h (Fig. 6A). Western blotting analysis revealed that as the LPS concentration increased, the p-p65 level increased significantly, whereas the p65 level did not increase significantly (Fig. 6B, C). Moreover, the expression of downstream inflammatory factors (IL-1β, TNF-α, and IL-6) also markedly and gradually increased as the LPS concentration increased (Fig. 6D). At the same time, we detected the protein level expression changes of StAR, CYP11A1, and AMH in LPS-treated KGN cells and control group by Western Blot. The accumulation levels of E2, FSH, and AMH in the cell culture supernatant were detected by ELISA. As shown in the new figure, LPS treatment significantly downregulated the protein expression of AMH, StAR and CYP11A1 (Fig. 6E, F). Meanwhile, ELISA detection showed that the expression of AMH and E2 in the LPS-stimulated group was significantly inhibited, and FSH levels increased (Fig. 6G). In the classic NF-κB pathway (Fig. 6H), LPS induces p65 nuclear translocation via the membrane receptor TLR4, thereby regulating the transcription of pro-inflammatory genes^32–34^. The downstream pathway activated by LPS via TLR4 was investigated by knocking down TLR4 expression with short hairpin RNA (shRNA) and by using TLR4 inhibitors. Knocking down the TLR4 gene or inhibiting TLR4 protein expression significantly attenuated the changes in downstream p-p65 protein levels induced by LPS (Fig. 6I, J). Nuclear-cytoplasmic separation and immunofluorescence experiments revealed that knocking down the expression of TLR4 significantly reduced the degree of p65 nuclear translocation induced by LPS (Fig. 6K, L).

### BME significantly ameliorates intestinal inflammatory injury induced by sucralose

**Fig. 7:**
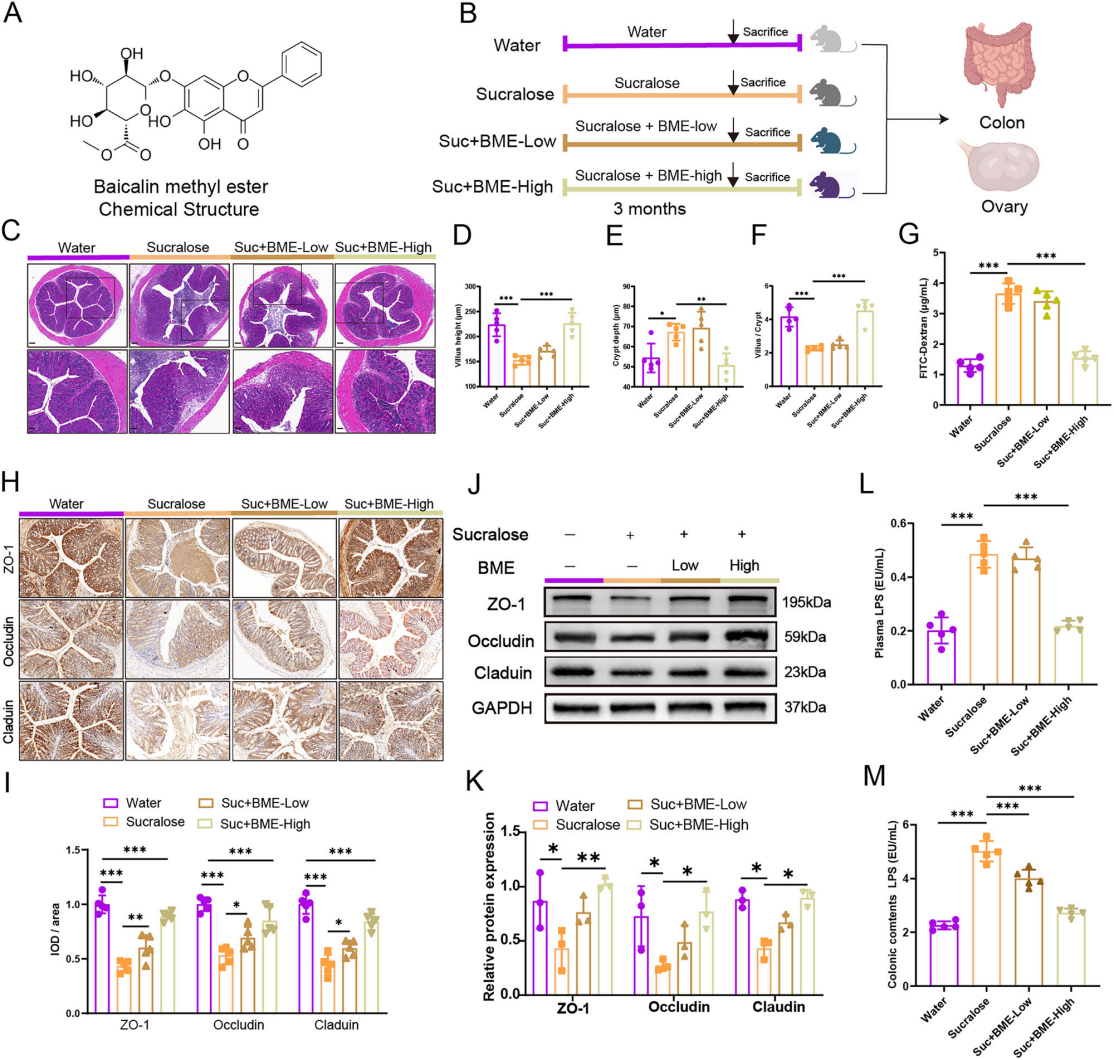
BME ameliorates intestinal inflammatory injury induced by sucralose. **A** Chemical structure of baicalin methyl ester (BME). **B** Design schematic representation of BME’s dose-dependent amelioration of sucralose-induced toxicity in mice. **C** Representative H&E staining images of intestinal sections from the four groups of mice. Scale bars = 100 μm (up) and 50 μm (down). **D**–**F** Comparison of villi height, crypt depth, and villi height/crypt depth in intestine samples of four groups (*n* = 5 per group). **G** The plasmatic concentration of FITC-dextran from the four groups of mice. **H**, **I** IHC-staining images and analyses of ZO-1, Occludin, and Claudin-1 proteins in intestinal tissues from the four groups of mice (*n* = 5 mice per group). Scale bars = 50 μm. **J**, **K** Western blot bands and relative protein expressions of ZO-1, Occludin, and Claudin-1 in intestinal tissues (*n* = 3 mice per group). **L**, **M** Concentration of LPS in plasma and colonic contents from the four groups of mice (*n* = 5 mice per group). The data represent the mean ± SEM. *p < 0.05, **p < 0.01, and ***p < 0.001.

Several studies have shown that baicalein (Fig. 7A) can alleviate LPS-induced tissue inflammation by blocking LPS-induced damage to the intestinal barrier in mice^25,35–37^. Moreover, some studies have reported that baicalein improves the aging of mouse granulosa cells and ovarian function through the mTOR signaling pathway^38–40^. As a result, we evaluated the protective effects of different concentrations of BME (a baicalin methylated derivative with enhanced bioavailability) on sucralose-induced colonic and ovarian inflammation in mice (Fig. 7B). The H&E staining analysis revealed that relative to the mice in the control group, the mice in the high-concentration BME treatment group had a greater villus height but considerably lower crypt depth in the small intestine, which increased the villus-to-crypt ratio (Fig. 7C–F). Compared with the sucralose group, the FITC glucan level in the BME-high treatment group was significantly decreased (Fig. 7G). Moreover, IHC analysis showed that tight junction protein levels were significantly higher in the BME-high treatment group than in the control group (Fig. 7H, I). Western blotting analysis confirmed that the intensity of expression of the abovementioned proteins was obviously increased in the tissue sections of the BME-high treatment group (Fig. 7J, K). We further measured LPS levels in mouse plasma and colon contents. The LPS levels were significantly lower in the BME-high treatment group relative to the sucralose group (Fig. 7L, M).

### BME significantly ameliorates sucralose-induced ovarian aging

**Fig. 8:**
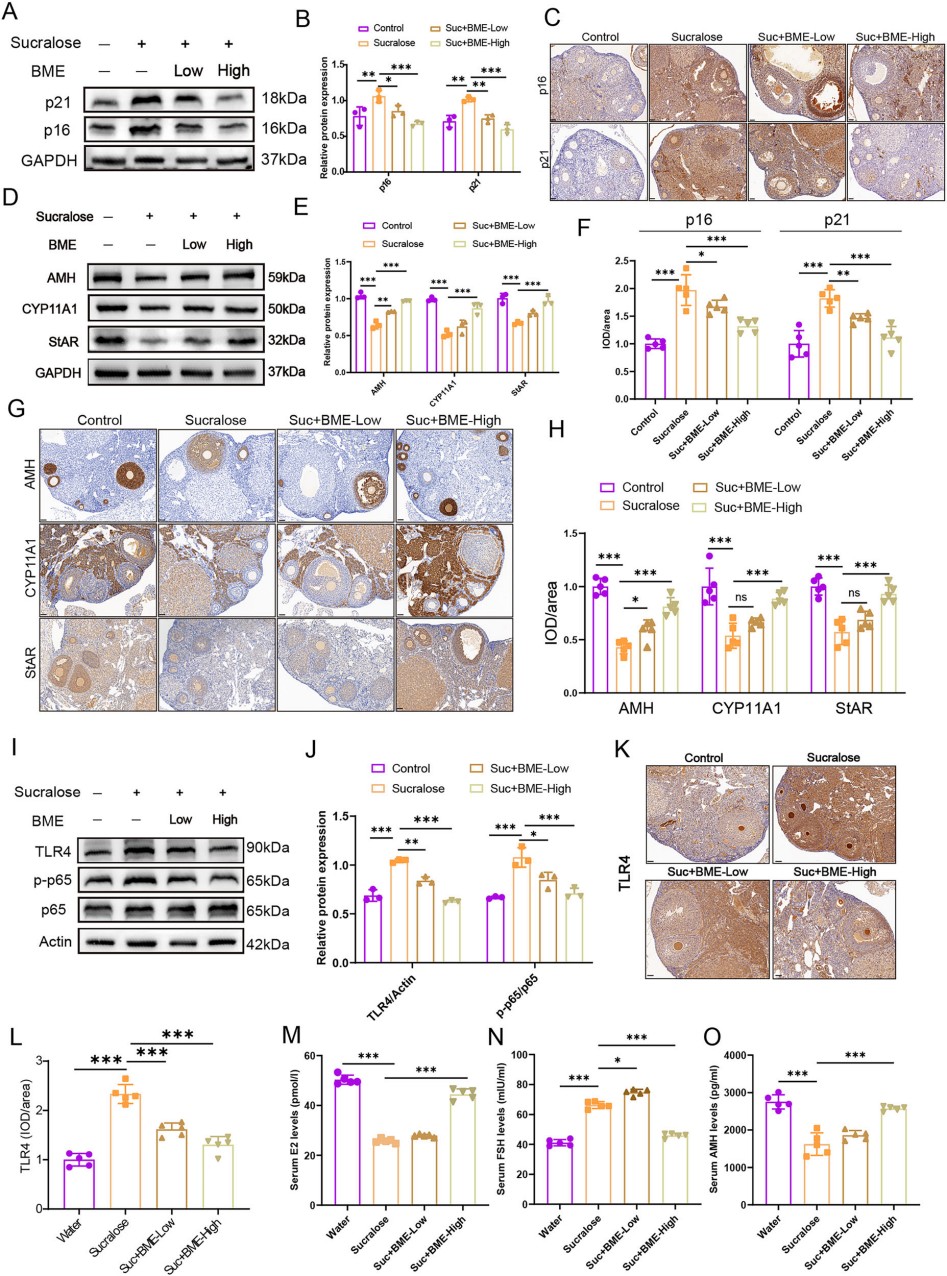
BME ameliorates sucralose-induced ovarian aging. **A**, **B** Western blot bands and relative protein expressions of p21, and p16 proteins in ovarian tissues. **C**, **F** IHC-staining images and analyses of p21, and p16 proteins in ovaries tissues (*n* = 5 mice per group). Scale bars = 50 μm. **D**, **E** Western blot bands and relative protein expressions of AMH, CYP11A1, and StAR in intestinal tissues. **G**, **H** IHC-staining images and analyses of AMH, CYP11A1, and StAR in ovaries tissues (*n* = 5 mice per group). Scale bars = 50 μm. **I**, **J** Western blot bands and relative protein expressions of TLR4, p-p65, and p65 proteins in ovarian tissues. **K**, **L** IHC-staining images and analyses of TLR4 in ovaries tissues (*n* = 5 mice per group). Scale bars = 50 μm. **M**–**O** Serum concentrations of FSH, E2, and AMH in the four groups of mice (*n* = 5 mice per group). The data represent the mean ± SEM. *p < 0.05, **p < 0.01, and ***p < 0.001.

Mice receiving sucralose with high-dose BME supplementation presented significantly greater ovarian weights and ovarian indices than those receiving sucralose alone (Fig. S4A–C). According to H&E staining analysis, the number of antral follicles in the high-dose BME + sucralose group was substantially greater than that in the sucralose-only group (Fig. S4D, E). Western blotting (Fig. 8A, B) and IHC (Fig. 8C, F) analyses revealed that ovarian tissue levels of p21 and p16 were significantly lower in the low-dose and high-dose BME + sucralose groups than in the sucralose-only group. Western blotting (Fig. 8D, E) and IHC (Fig. 8G, H) analyses also revealed significantly higher levels of CYP11A1, StAR, and AMH in ovarian tissues in the high-dose BME + sucralose group than in the sucralose-only group. Western blotting analyses (Fig. 8I, J) revealed that the ovarian tissue levels of TLR4/GAPDH and p-p65/p65 were significantly lower in the low-dose and high-dose BME + sucralose groups than in the sucralose-only group. IHC analyses (Fig. 8K, L) also revealed significantly lower TLR4 levels in the low-dose and high-dose BME + sucralose groups. Consistent with this finding, the serum AMH and E2 concentrations were significantly higher, and the FSH levels were lower in the high-dose BME + sucralose group than in the sucralose-only group (Fig. 8M–O).

## Discussion

Sucralose, a high-intensity artificial sweetener (600 times sweeter than sucrose) that contributes negligible calories due to limited human metabolism, has been increasingly incorporated into beverages, dairy products, baked goods, pharmaceuticals, and health supplements in recent years. While the global usage of sucralose continues to increase, the potential consequences of artificial sweeteners for the gut microbiota composition and metabolic health remain incompletely understood. This study provides evidence that long-term sucralose exposure is accompanied by gut microbiota dysbiosis, impaired intestinal barrier integrity, increased gut-derived LPS, and ovarian inflammatory activation. Importantly, our FMT experiments show that transferring fecal microbiota from sucralose-exposed donors to recipient mice is sufficient to reproduce key intestinal injury, elevated LPS, and ovarian aging-related phenotypes, supporting a microbiome-associated and transmissible component of the observed effects. Nevertheless, because we did not directly profile the microbiome of post-FMT recipient mice, we cannot determine which microbial taxa, community features, or functional pathways are responsible for phenotype transmission. Future studies incorporating post-FMT microbiome profiling (e.g., 16S rRNA sequencing) will be required to define the specific microbial drivers and strengthen mechanistic causality. Circulating LPS binds to TLR4 on the surface of ovarian granulosa cells, activating the NF-κB signaling pathway. LPS not only induces KGN cells to produce inflammatory factors (such as IL-6, TNF-α), but also directly inhibits the key initiating step of steroidogenesis (StAR expression) and follicle reserve marker (AMH). This provides a more direct molecular mechanism explanation for inflammation related infertility, such as endometriosis and pelvic inflammatory disease, where inflammatory signals simultaneously disrupt the endocrine function (hormone synthesis) and paracrine regulation function (AMH signaling) of granulosa cells. This activation also promotes nuclear translocation of the p65 subunit and upregulates the transcription of downstream inflammatory cytokines. This cascade leads to a decrease in the ovarian reserve and endocrine dysfunction. Our findings suggest that the gut microbiota–metabolite–ovary axis may play an important contributory role in the adverse impacts of sucralose on ovarian function. Targeted interventions modulating this axis may have therapeutic effects and ameliorate sucralose-induced ovarian dysfunction. These discoveries pave the way for novel intervention strategies aimed at preserving ovarian health in the context of widespread exposure to food additives.

Research on sucralose suggests that it may adversely affect multiple organ systems by inducing inflammatory responses, particularly under conditions of high-dose or chronic exposure, raising concerns about its health hazards^2^. Sucralose may activate the membrane receptor T1R3, leading to the production of reactive oxygen species (ROS), increased lipogenesis, and endoplasmic reticulum (ER) stress, thereby accelerating the progression of hepatic steatosis^41^. Other studies have reported markedly elevated high-density lipoprotein (HDL) and total cholesterol levels following sucralose consumption^42^. Some studies have found an association between sucralose intake and an increase in the risk of cardiovascular mortality, with this risk being amplified in overweight and obese people^43^. Yao et al. reported that mice exposed to sucralose had disrupted estrous cycles, characterized by a reduction in the number of primordial, primary, and secondary follicles, as well as a large number of blocked follicles^44^. Accompanying this is a decrease in the serum FSH, E2, and progesterone (P4) levels, resulting in a significant increase in the LH/FSH ratio^44^. Sucralose can also induce insulin resistance, manifested by an increase in serum insulin levels and impairment in insulin resistance^44^. However, this study has a key limitation in that it lacks a comprehensive investigation into the underlying molecular mechanisms and, consequently, the absence of therapeutic strategies developed based on these mechanisms. Substantial evidence indicates that the consumption of sucralose is associated with gut dysbiosis. Research on the intricate crosstalk between gut dysbiosis and various organ systems has intensified in recent years. Thus, this microbial imbalance can affect ovarian function through multiple pathways, including inflammation, immune dysregulation, hormonal disruption, and metabolic alterations. These effects promote follicular apoptosis and foster a pro-inflammatory microenvironment in the ovary, potentially contributing to the onset or exacerbation of primary ovarian insufficiency. We added sucralose to the drinking water provided to female mice to explore this issue. Our findings suggest that long-term use of sucralose may affect women’s reproductive health by affecting inflammation or metabolism. Sucralose may induce intestinal inflammation in mice. This inflammation manifests as crypt destruction, loss of surface epithelial cells and goblet cells, and an increase in inflammatory cell infiltration, concurrent with dysbiosis of the gut microbiota, dysregulated digestive protease activation, and impaired integrity of the intestinal barrier. The intestinal barrier serves as the interface between the intestinal lumen environment and the intestinal immune system and exerts a vital role in maintaining health and disease occurrence^45,46^. The integrity of the intestinal epithelial barrier is crucial for maintaining the healthy coexistence of epithelial cells with intestinal bacteria, whereas tight junction proteins are essential for maintaining barrier integrity^47^. Compromised intestinal barrier integrity allows gut-derived LPS to translocate into the systemic circulation, thereby triggering chronic inflammation. Canonical and non-canonical NF-κB pathways: The IKK complex becomes activated upon stimulation by pro-inflammatory cytokines, growth factors, or LPS, thereby phosphorylating IκB proteins (e.g., IκBα and IκBβ)^48^. Subsequently, these phosphorylated IκB proteins are ubiquitinated and degraded by the proteasome, liberating the NF-κB complex^49^. Activated p65 translocates into the nucleus to directly bind to and promote the transcription of multiple genes encoding inflammatory mediators^50^. This results in elevated levels and the release of such inflammatory factors, thereby contributing to local ovarian inflammation and associated pathophysiological changes^50–52^.

To elucidate the mechanisms underlying ovarian dysfunction, we performed transcriptome sequencing analysis on ovarian tissues from control and sucralose-treated mice. Our findings indicated that the activation of inflammatory pathways, which include the NF-κB pathway, represents a primary pathological feature in the ovaries of the sucralose-exposed mouse model. However, the precise mechanisms governing the interplay between the gut microbiota and ovarian function remain elusive. A major source of endotoxin (LPS) is gram-negative enteric bacilli, which reside in the gut^53^. Disruption of intestinal epithelial tight junction compromises barrier function, permitting the translocation of bacterially released LPS into the circulation and consequently increasing systemic LPS levels^54,55^. Several studies have indicated that sucralose adversely affects ovarian function and other organ systems. Hsia et al. demonstrated that sucralose impairs autophagy, induces oxidative stress, and causes DNA damage, thereby negatively affecting male reproductive health in mice^56^. Lu et al. reported that long-term sucralose intake disrupts FXR signaling activation, disturbing hepatic lipid and cholesterol homeostasis, probably via the ability of the gut microbiota to reduce bile acid metabolism^57^. Cao et al. reported that maternal sucralose consumption alters the maternal microbiota balance, subsequently modifying the offspring gut microbiota and exacerbating hepatic steatosis in adulthood^58^. Our study also demonstrated the deleterious impacts of long-term sucralose intake on ovarian function and elucidated the underlying mechanisms, thereby contributing to the understanding of the gut microbiota-ovary axis. However, this study has several inherent limitations. Firstly, we could not identify the bacterial species responsible for an increase in LPS production. This limitation stems primarily from the low resolution of 16S rRNA gene sequencing for species-level detection and the complex origins of LPS in the gut. Secondly, clinical validation of our proposed mechanisms and therapeutic outcomes was not performed. Thirdly, this study used 0.1 mg/ml of sucralose drinking water for intervention. In view of this, the concentration is much higher than the sweetness of conventional human consumption beverages. It is inappropriate to directly equate this concentration with the sweetness of human beverages. Our experimental design aims to ensure sufficient biological exposure levels are achieved in animal models. Fourthly, regarding the intervention measures, we referred to the previously established pharmacological protocols in this field for the BME dosage^25^, which ensures the reliability of the starting point of the experiment, but also means that we have not yet depicted its dose-response curve. This is a direction that needs to be improved in future research. Fifthly, an important limitation of this study is that the microbiome of post-FMT recipient mice was not directly profiled. Although FMT demonstrates transmissibility of the phenotype from sucralose-exposed donors to recipients, post-FMT microbiome characterization will be essential to confirm engraftment patterns and to identify specific taxa and functional pathways responsible for the transferred effects. Finally, the present study does not definitively prove that TLR4 signaling is the central pathway for BME’s action in vivo. More specific approaches, such as the use of TLR4-selective antagonists or knockout models, will be essential in future work to rigorously test this hypothesis. Therefore, we could not provide robust clinical evidence to support translational research. Fourthly, regarding the intervention measures, we referred to the previously established pharmacological protocols in this field for the BME dosage, which ensures the reliability of the starting point of the experiment, but also means that we have not yet depicted its dose-response curve. This is a direction that needs to be improved in future research.

## Methods

### Animal treatment and sampling

The Committee of Animal Care and Utilization of Tongji University (TJBG04025101) approved our animal experimental protocol. C57BL/6 female mice (Shanghai Model Organisms; 8 weeks old) were classified into two groups at random: the control and sucralose treatment groups. The mice were kept in an experimental animal feeding center under a 12-h/12-h light/dark cycle with constant humidity and temperature. Both groups were provided with an identical standard diet (10% of total energy derived from fat, 20% from protein, and 70% from carbohydrates; H10010, Research Diets, Beijing, China). For treating the mice, 0.1 mg/mL sucralose (HY-N0614, MedChemExpress, USA) was added to the drinking water, which is equal to the upper limit of acceptable daily intake (ADI) of human beings approved by the FDA (5 mg/kg/day) and was used in our previous study^44,57,58^. The BME treatment groups, which were divided into low or high (0.25 or 1.0 mg/mL) groups, received BME co-dissolved with sucralose in the drinking water^25^. During the experiment, all animals were fed with standard rodent feed and were fed freely. Drinking water is also freely ingested. After 3 months of treatment, the mice were euthanized via carbon dioxide, and tissue samples were collected. The weights of the mice in both groups were measured once a week. After treatment, blood was collected, the ovaries were dissected, and their weights were measured. One part of the ovarian tissue was retained for protein extraction, whereas the rest was fixed overnight with 4% paraformaldehyde before the experiments. All mice were euthanized during the specific stage of pre-estrus to collect ovarian tissue and blood samples, thereby eliminating the impact of estrus cycle fluctuations on histological and hormone measurement results.

### Cell culture and treatment

We obtained KGN cells from the RIKEN BioResource Research Center (RCB1154; Japan). These cells were cultured in DMEM/F-12 medium (G4613, Servicebio, China) supplemented with 10% fetal bovine serum (209111, NEST, China) and 1% penicillin-streptomycin (abs9244, Absin, China). The medium was replaced on alternate days. These cells were later grown in a sterile incubator at 37 °C with 5% CO_2_. Then, KGN cells were stimulated with 1 μg/mL LPS (purified *E. coli* LPS; KGR0048; KeyGEN Bio-TECH; China) as an inflammatory stimulus for 24 h. To downregulate the expression of TLR4, the cells were pretreated with TAK-242 (selective TLR4 inhibitor, MedChemExpress) and then with LPS for 24 h.

### Western blotting analysis

Mouse organ tissue samples were lysed in an appropriate amount of RIPA buffer (abs9229, Absin, China). The protein content was quantified with a BCA protein assay kit, while Omni Easy™ protein sample loading buffer (LT101, Epizyme Biomedical Technology Co., Ltd., China) was used to prepare protein solutions of the same concentration. An equal amount of total protein (20 μg) per well was separated by 10% SDS-PAGE. Electrophoresis was performed at 80 V for 120 min, after which the protein was transferred onto a PDVF membrane with a pore size of 0.45 μm (IPVH00010, Millipore, USA). After being blocked with skim milk (abs9175, Absin, China) for 1 h, the proteins were incubated with different primary antibodies overnight at 4 °C. After rinsing with TBST three times, HRP-labeled goat anti-rabbit IgG (H + L) (AS014, ABclonal, China) was added, and the proteins were subject to incubation for 2 h at ambient temperature. After rinsing with TBST, immune response bands were observed and detected using an Omni-ECL™ Femto Light chemiluminescence kit following specific protocols (SQ201L, Epizyme Biomedical Technology Co., Ltd., China). Then, the samples were analyzed using an automated ECL image analysis system (5200, Tanon, China). The loading control (GAPDH or β-tubulin) is from the same membrane which was cut prior to antibody incubation. The primary antibodies used are shown in Table S1.

### Serological assays

Blood samples were centrifuged at 4 °C to separate and collect the serum and the plasma. Enzyme-linked immunosorbent assay (ELISA) kits were adopted for detecting follicle-stimulating hormone (FSH) (BYabscience; cat#: BY-JZF1604), 17β-estradiol (E2) (BYabscience; cat#: BY-JZF0048), and anti-Mullerian hormone (AMH) (BYabscience; cat#: BYHS500492) in the serum samples. ELISA kits were also used to test LPS (BY-JZF1158, BYabscience) in colon contents and plasma samples.

### Quantitative real-time PCR analysis (RT-qPCR)

Total cellular and tissue RNA were isolated using total RNA extraction reagent (RC102, Vazyme, China) for qPCR and later reverse-transcribed into cDNA using ABScript III Reverse Transcriptase (RK20408, ABclonal, China). Next, RT-PCR was performed with Genious 2X SYBR Green Fast qPCR Mix (RK21203, ABclonal, China). A 10-μL reaction system was prepared, containing 200 nM primers, SYBR Green Fast qPCR Mix (5 μL), template cDNA (1 μL), and nuclease-free water. PCR amplification was performed as follows: 3 min of denaturation at 95 °C; 5 s of amplification at 95 °C and 34 s at 65 °C for 40 cycles; and melting curing. β-actin served as a reference gene. The information on primers is provided in Table S2.

### Ovarian histology and follicular counting

After 24 h of fixation with 4% paraformaldehyde, the ovarian tissues were subjected to gradient dehydration with alcohol and xylene, followed by embedding in paraffin. Then, they were cut into sections (5 μm thick). These ovarian sections were dewaxed, rehydrated with xylene and ethanol, and exposed to hematoxylin and eosin (H&E) staining. Finally, the number of follicles in the sections in the two groups was determined via optical microscopy.

### Immunohistochemistry (IHC)

Formalin-fixed ovarian tissues were prepared in paraffin sections, followed by xylene dewaxing. Next, the sections were incubated for 15 min with 3% hydrogen peroxide for antigen repair. After blocking with goat serum (10% FBS-TBST) for 30 min, the sections were subject to incubation with antibodies. They were later sealed before being observed and photographed under a microscope.

### Immunofluorescence (IF)

The paraffin-embedded tissue sections were dewaxed and dehydrated with a gradient of alcohol. After antigen repair, the sections were blocked with goat serum (10% FBS-TBST) for 30 min and incubated with primary antibodies overnight. The following day, the secondary antibodies were added and incubated for 30 min. Regarding the cell immunofluorescence experiments, we inoculated the cell suspension onto glass coverslips (801010, NEST, China) for another 24 h of treatment. After blocking, these cell climbing sheets were incubated with primary and secondary antibodies in succession and observed with a fluorescence microscope.

### Fecal microbiota transplantation (FMT)

Fecal microbiota transplantation (FMT) was performed as previously described^59–62^. Briefly, fresh mouse fecal samples were collected, mixed with phosphate-buffered saline, and passed through a 200 µm sieve to remove large particles. The mixture was vortexed and centrifuged at 1000 × *g* (4 °C) for 5 min, after which the supernatant was gathered. Additionally, it was thoroughly mixed with 20% glycerol at a 1:1 ratio, followed by immediate freezing in liquid nitrogen and storage at –80 °C. Before the samples were used, they were diluted with physiological saline to 50 mg/mL and later passed through a 70 μm cell sieve. Before undergoing FMT, the mice received an antibiotic water treatment (1 g/L ampicillin, 0.5 g/L vancomycin, 0.1 g/L gentamicin, 0.5 g/L neomycin, and 0.01 g/L erythromycin) for 2 weeks to deplete the existing gut microbiota. All mice that underwent FMT were orally administered 100 μL of filtrate every 2 days for 8 weeks.

### RNA sequencing (RNA-seq)

To further investigate the mechanism by which sucralose affects ovarian aging, we conducted comprehensive transcriptome sequencing. This sequencing experiment set up a control group and a sucralose group, each containing 3 mouse ovarian samples, and each sample was derived from an independent individual, all of which were biological replicates. Total mouse ovarian RNA was extracted (RC102, Vazyme, China) to construct RNA libraries through the NEBNext Ultra RNA Library Prep Kit for Illumina (New England Biolabs). The Illumina NovaSeq 6000 platform was used to sequence cDNA libraries. After sequencing, FastQC was employed to perform quality control on the data to remove low-quality reads. HISAT2 software (version 2.2.4) was adopted for aligning the remaining clean reads with the reference genome. All RNA-seq libraries exhibited high and comparable sequencing quality across the control and sucralose groups, with ~46–67 million clean reads per sample, >99% clean reads and >93% bases above Q30 (Table S3). The thresholds for identifying differentially expressed genes (DEGs) in the two groups included |fold change | > 1.5 and FDR < 0.05. We used the *p*-adjust function (Benjamini-Hochberg method) in the DESeq2 R package for FDR correction. Only DEGs meeting the criteria of |fold change | > 1.5 and FDR < 0.05 were used as input for KEGG pathway enrichment analysis. GSEA was likewise conducted using the DEG set derived from the same FDR-controlled differential expression analysis. The data were explored and visualized in R (version 4.2.1). GSEA 4.3.2 was used for gene set enrichment analysis (GSEA) with MSigDB database-derived hallmark gene sets.

### Sequencing of the 16S rRNA gene and data analysis

In total, ten samples from every group (20 total) were used for 16S rRNA sequencing. Microbial community profiling was performed via high-throughput sequencing of the hypervariable V3–V4 regions of the 16S rRNA gene. The V3–V4 region of the bacterial 16S rRNA gene was amplified using general primers (341 F: 5’-ACTCCTACGGGAGGCAGCAG-3’, and 806 R : 5’-GGACTACHVGGGTWTCTAAT-3’)^63,64^. Genomic DNA was extracted from the colonic contents using a Qiagen gel extraction kit. A TruSeq® DNA PCR-Free Sample Preparation Kit (Illumina, USA) was adopted for generating sequencing libraries following specific protocols. The integrity and content of DNA were assessed by 1% agarose gel electrophoresis and Qubit fluorometry. The amplification products were sequenced on the MiSeq platform (Illumina, USA). We used the DADA2 plugin for its quality filtering, denoising, and read-merging functions (the dada2 denoise-paired command in QIIME2). However, the final feature table was generated by clustering the resulting representative sequences into Operational Taxonomic Units (OTUs) at a 97% similarity threshold using the VSEARCH clustering tool within QIIME2. The taxonomy of each OTU representative sequence was analyzed by RDP Classifier version 2.2 against the 16S rRNA database (e.g., Silva v138) using confidence threshold of 0.7. The alpha diversity indices (including the ACE, Chao1, Shannon, and Simpson indices) of the samples in the groups were analyzed via bioinformatics. A web-based platform (https://huttenhower.sph.harvard.edu/galaxy/) for linear discriminant analysis effect size (LEfSe) analysis was used to determine typical species.

### Assessment of estrous cycles

The estrous cycle was monitored daily for twelve consecutive days. According to the previous method^54,65^, estrous cycle stages, including diestrus (D), metestrus (M), estrus (E), and proestrus (P), were identified by the types of vaginal epithelium cells of mice. Vaginal epithelial cells were collected by flushing the vagina with sterile saline and stained with 0.1% methylene blue. Cytological features were examined under a light microscope to determine the stage of the estrous cycle.

### Determination of in vivo permeability of FITC-dextran

As mentioned previously^66,67^, Fluorescein isothiocyanate (FITC)-conjugated dextran 4 kDa (HY-128868A, MedChemExpress) was used. All mice were fasted for 12 h. The FITC-dextran (150 mg/kg) was dissolved in sterile phosphate-buffered saline (PBS) and then administered to the mice via oral gavage. Four hours later, the mice were euthanized and peripheral blood was collected and kept in the dark for 30 min. Samples were centrifuged at 4 °C for 10 min at 1500 × *g* to collect serum. The fluorescence intensity of the serum was measured, and the concentration of FITC-dextran in the serum was calculated using a standard curve. A standard curve was generated by serially diluting a 10 mg/mL stock solution of FITC-dextran in PBS. Fluorescence intensity was measured using a FlexStation 3 (Molecular Devices, San Jose, CA) at an excitation wavelength of 485 nm and an emission wavelength of 528 nm.

### Statistical analysis

Each experiment was independently repeated three times. The data are presented as mean ± standard error (SEM). Use Shapiro–Wilk test and Levene test to evaluate the normality and homogeneity of variance of the data, respectively. If normality and homogeneity of variance are satisfied, parameter testing is used; If there is a significant deviation, non-parametric testing should be used. The key data of this study all meet the premise of parameter testing. The comparison between the two groups was conducted using the two-tailed non-paired Student’s *t* test; When comparing multiple groups, if the parameter conditions are met, use one-way ANOVA; otherwise, use Kruskal–Wallis test. If ANOVA shows significant main effect, Tukey’s HSD test will be used for post hoc pairwise comparisons to control for overall error rate. The significance threshold is set to *p* < 0.05. All statistical analyses were conducted using GraphPad Prism 8.0.2.

## Supplementary information

Supplementary Information

## Data Availability

The raw 16S rRNA gene sequencing reads and associated metadata generated in this study have been deposited in the NCBl SRA under the BioProject accession number PRJNA1380064. All data are available from the corresponding authors upon request.
