# Supplementary material for: Long-term sucralose exposure accelerates ovarian aging via gut microbiota dysbiosis
Source: NPJ Sci Food. 2026 Mar 24;10:229. doi: 10.1038/s41538-026-00775-x (PMC13400739; doi:10.1038/s41538-026-00775-x)
Supplement: Supplementary file 1 — Supplementary Information [file 41538_2026_775_MOESM1_ESM.docx]

**Long-term sucralose exposure accelerates ovarian aging via gut microbiota dysbiosis**

Junfeng Chen^a^, Donghai Zhang^a^, Jie Gao^a^, Ziyi Zhang^a^, Junjie Qu^b^, Yiran Li^a,b,^ *

^a^Shanghai Key Laboratory of Maternal Fetal Medicine, Shanghai Institute of Maternal-Fetal Medicine and Gynecologic Oncology, Shanghai First Maternity and Infant Hospital, School of Medicine, Tongji University, Shanghai, China.

^b^Center for Reproductive Medicine, Shanghai First Maternity and Infant Hospital, School of Medicine, Tongji University, Shanghai, China.

*Corresponding authors:

Shanghai First Maternity and Infant Hospital, School of Medicine, Tongji University, Shanghai, China.

Dr. Yiran Li; [liyiran2007@gmail.com](mailto:liyiran2007@gmail.com)


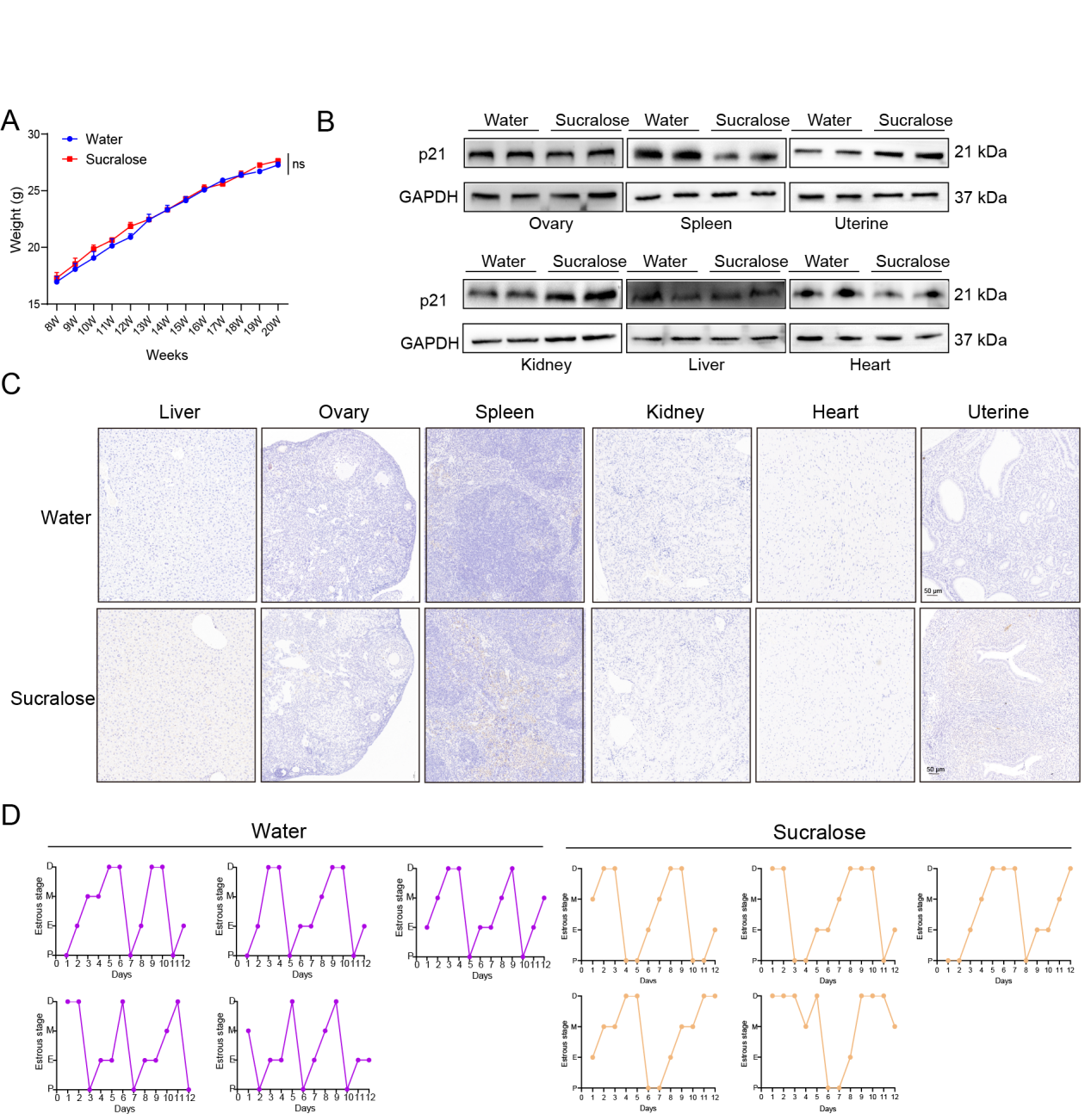


**Fig. S1. Short-term sucralose intake did not significantly induce multi-organ aging.** (A) Body weight changes in the water-treated and long-term sucralose-treated throughout the experimental period. (B) Western blot bands of the senescence marker p21 in ovaries and other organs from water-treated and short-term sucralose-treated mice. (C) Representative IHC staining of the senescence marker p21 in ovaries and other organs from water-treated and short-term sucralose-treated mice. Scale bar = 50μm. (D) Estrus cycles in female mice receiving normal drinking water group (n=5 mice per group) and drinking water group containing sucralose (n=5 mice per group).

**
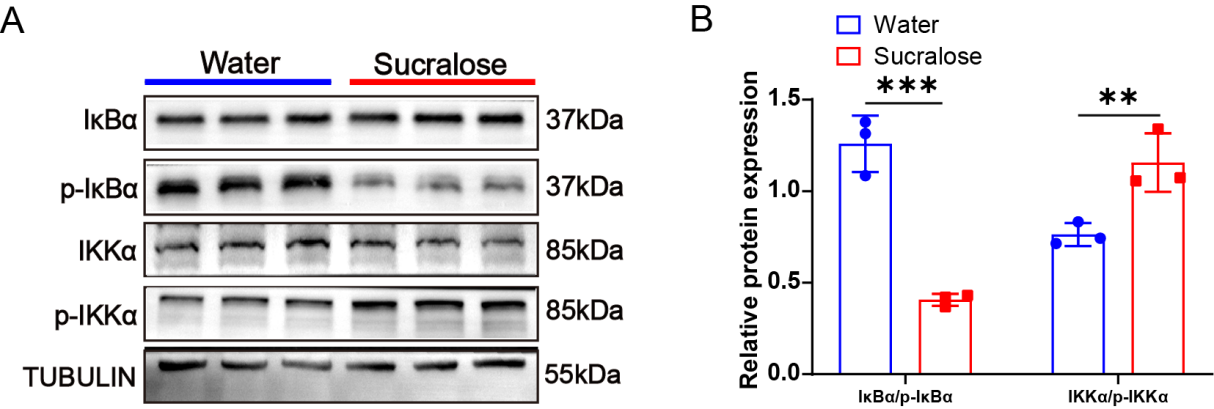
**

**Fig. S2.** Western blot images and relative protein expression levels showed protein expression levels of IκBα, p-IκBα, IKKα, and p-IKKα in ovarian tissue (n = 3 mice per group). Data are represented as the mean ± SEM. ***P* < 0.01, ****P* < 0. 001.

**
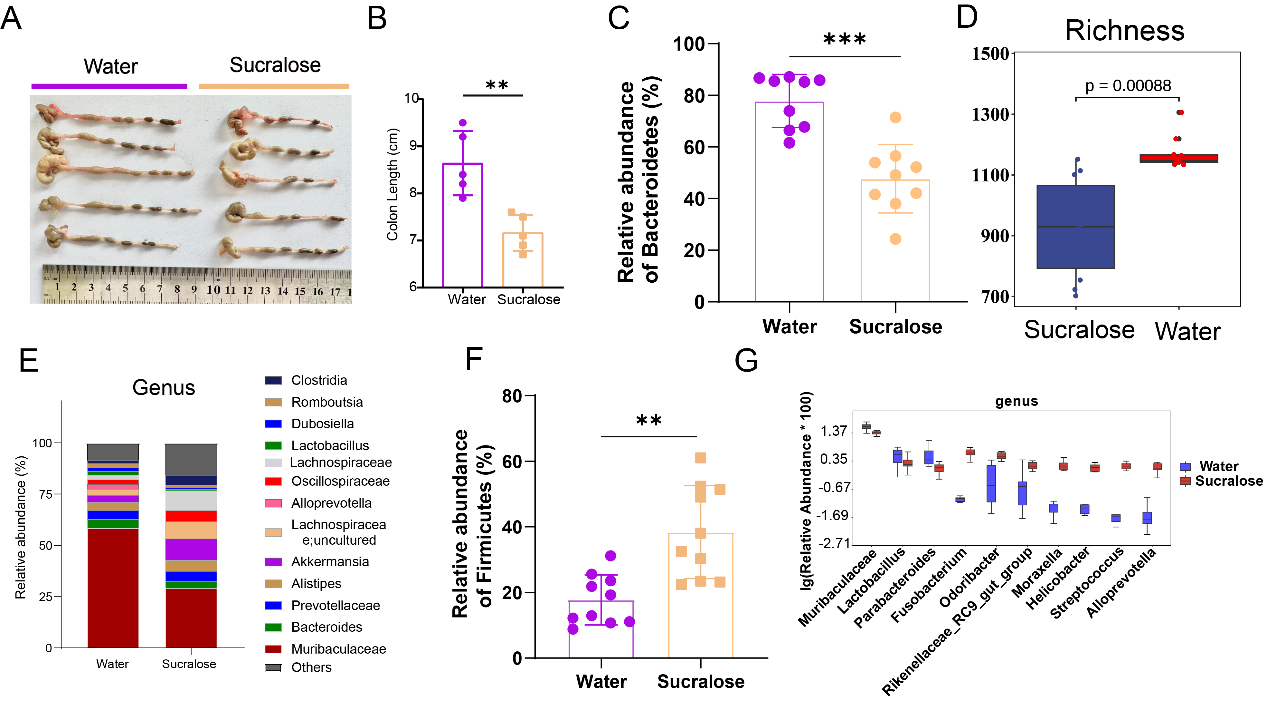
**

**Fig. S3. Alterations in the gut microbiota and intestinal environment induced by long-term sucralose consumption.** (A) Representative image of colons. (B) Average colon length in two groups. Data are shown as means ± SEM (n = 5 mice per group). (C, F) Comparison of the relative abundance of *Bacteroidetes* and *Firmicutes* in two groups. (D) Richness index was compared in two groups. (E) Relative abundance of microbiota at the genus level in two groups. (G) Boxplots illustrate the differentially abundant bacterial genera between the two groups. Data are represented as the mean ± SEM. ***P* < 0.01, ****P* < 0. 001.

**
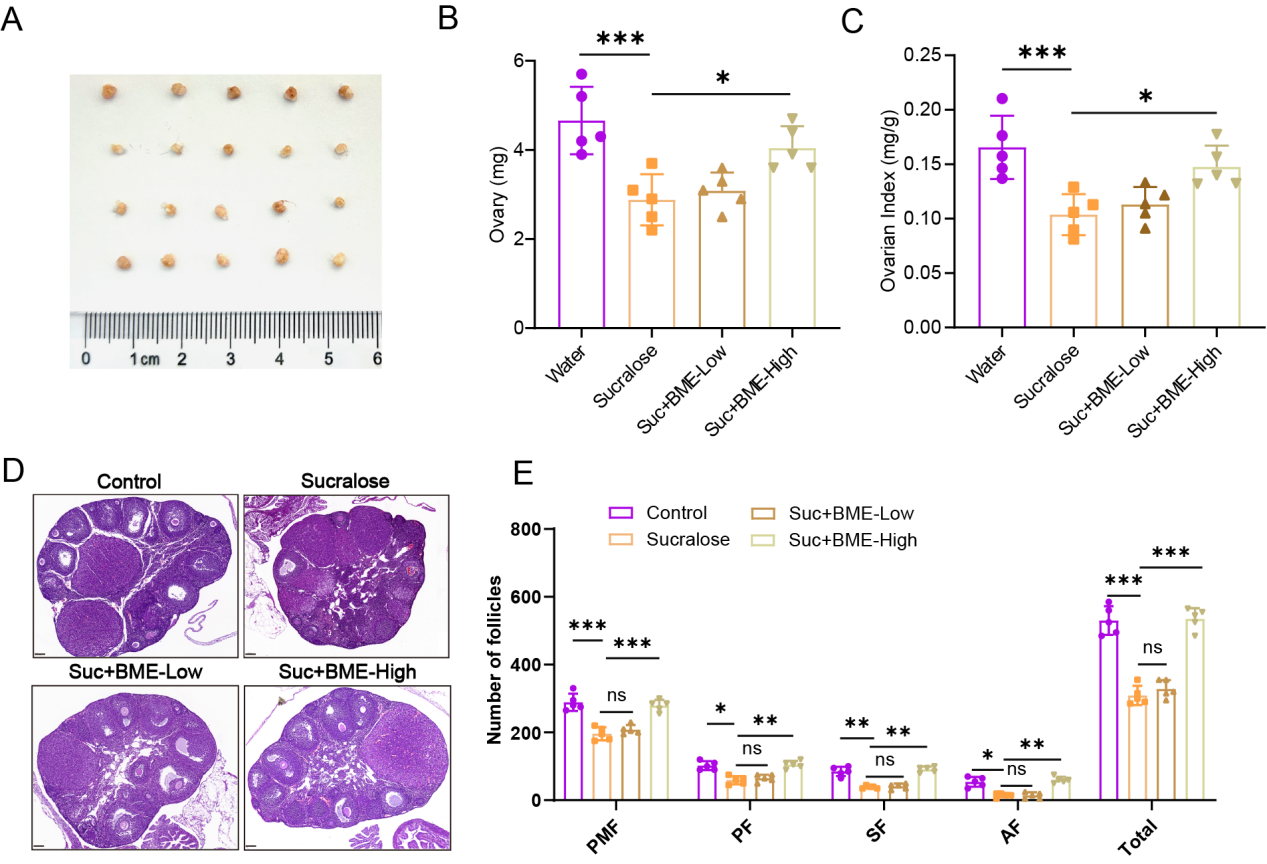
**

**Fig. S4. BME significantly alleviated the manifestations of sucralose-induced premature ovarian failure.** (A) Representative images of mouse ovaries from the four groups of mice. (B, C) Ovary weight and ovary index of four groups (n = 5 mice per group). (D) Representative H&E staining images of ovarian sections in the four groups of mice. Scale bars = 100 μm. (E) Numbers of ovarian follicles at different stages in the four groups of mice (n = 5 mice per group). Data are represented as the mean ± SEM. **P* < 0.05, ***P* < 0.01, ****P* < 0. 001.

**Table S1. The information of primary and secondary antibodies.**

| **Primary antibodies** | **Company and Cat.No.** |
| --- | --- |
| Occludin | Abcam |
|  | ab216327 |
| Claudin | Abcam |
|  | ab307692 |
| ZO-1 | Abcam |
|  | ab276131 |
| p21 | ABclonal |
|  | A19094 |
| p16 | Cell Signaling Technology (CST) |
|  | #23200 |
| p53 | ABclonal |
|  | A25915 |
| Lamin B1 | ABclonal |
|  | A11495 |
| AMH | Abcam |
|  | ab313767 |
| CYP11A1 | absin |
|  | abs174464 |
| StAR | absin |
|  | abs124642 |
| p65 | Abcam |
|  | Ab16502 |
| Phospho-p65 | Abcam |
|  | ab86299 |
| TLR4 | Proteintech |
|  | 19811-1-AP |
| MyD88 | ABclonal |
|  | A0980 |
| Lamin A/C | Proteintech |
|  | 10298-1-AP |
| IκBα | ABclonal |
|  | A1187 |
| Phospho-IκBα | ABclonal |
|  | AP0707 |
| IKKα | ABclonal |
|  | A2062 |
| Phospho-IKKα | ABclonal |
|  | AP0506 |
| β-Tubulin | ABclonal |
|  | A12289 |
|  |  |
|  |  |
| **Secondary antibody** | **Company and Cat.No.** |
| HRP-conjugated Goat anti-Rabbit IgG (H+L) | ABclonal |
|  | AS014 |

**Table S2. The primers for target genes and the sequences of shRNA.**

| **Genes** | **Primers (5’- 3’)** |
| --- | --- |
| β-actin | F: ACGCAGCTAGTAACAFTCC |
|  | R: AGATCAAGATCATTGCTCCCCT |
| IL-1β | F: GAAATGCCACCTTTTGACAGTG |
|  | R: TGGATGCTCTCATCAGGACAG |
| TNF-α | F: GTAGCCCACGTCGTAGCAAA |
|  | R: TTGAGATCCATGAAGTTGGC |
| IL-6 | F: GGGACTGATGCTGGTGACAA |
|  | R: ACAGGTCTGTTGGGAGTGGT |
|  |  |
|  |  |
| **The sequences of shRNA** | |
| sh-TLR4-nc | TTCTCCGAACGTGTCACGT |
| sh-TLR4-1 | CCGCTGGTGTATCTTTGAATA |
| sh-TLR4-2 | GCCACCTCTCTACCTTAATAT |
| sh-TLR4-3 | CGTTTGGTTCTGGGAGAATTT |

**Table S3. Summary of sequencing quality control metrics for RNA-seq samples.**

| Sample | NC1 | NC2 | NC3 | SUC_1 | SUC_2 | SUC_3 |
| --- | --- | --- | --- | --- | --- | --- |
| Raw reads | 48474914 | 46858630 | 61205374 | 67830236 | 55618984 | 58350006 |
| Raw read pairs | 24237457 | 23429315 | 30602687 | 33915118 | 27809492 | 29175003 |
| Raw bases | 7319712014 | 7075653130 | 9242011474 | 10242365636 | 8398466584 | 8810850906 |
| Clean reads | 48054602 | 46491294 | 60747700 | 67289078 | 55174542 | 57816650 |
| Clean bases | 7186527970 | 6954986937 | 9086946136 | 10067384577 | 8260028185 | 8647694242 |
| Clean read pairs | 24027301 | 23245647 | 30373850 | 33644539 | 27587271 | 28908325 |
| Average length | 149.5 | 149.6 | 149.6 | 149.6 | 149.7 | 149.6 |
| Clean reads % | 99.13% | 99.22% | 99.25% | 99.20% | 99.20% | 99.09% |
| Clean bases % | 98.18% | 98.29% | 98.32% | 98.29% | 98.35% | 98.15% |
| Q20 % | 97.70% | 97.90% | 97.95% | 98% | 97.95% | 97.90% |
| Q30 % | 93.95% | 94.40% | 94.50% | 94.55% | 94.40% | 94.30% |

**Source Data**

Figure 1 B


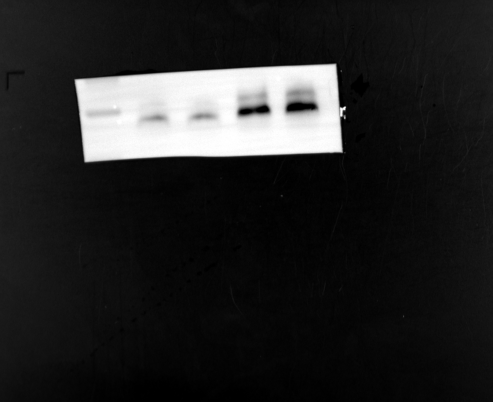

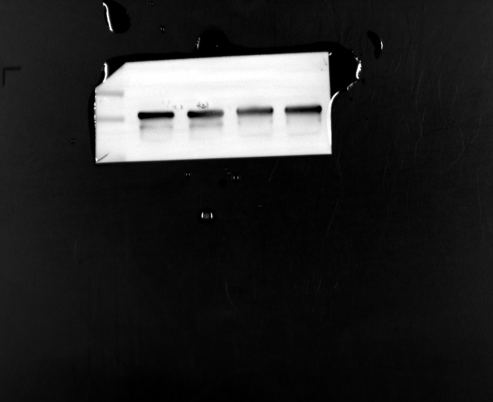


GAPDH

37kDa

p21

18kDa

Sucralose

Water

Sucralose

Water

Ovary


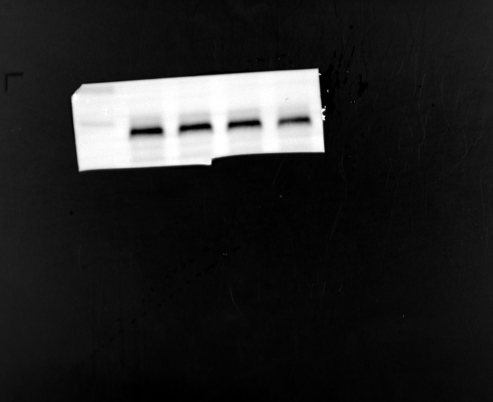

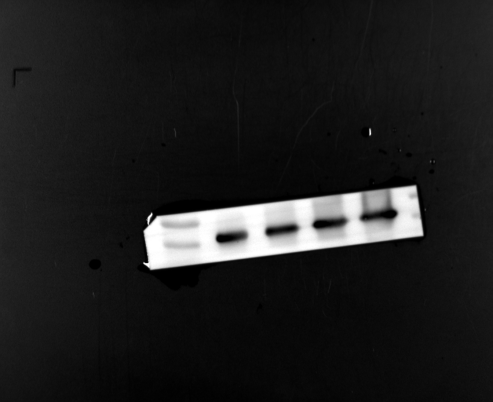


p21

18kDa

GAPDH

37kDa

Sucralose

Water

Sucralose

Water

spleen


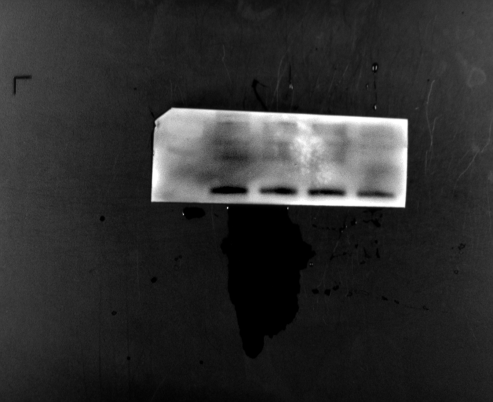

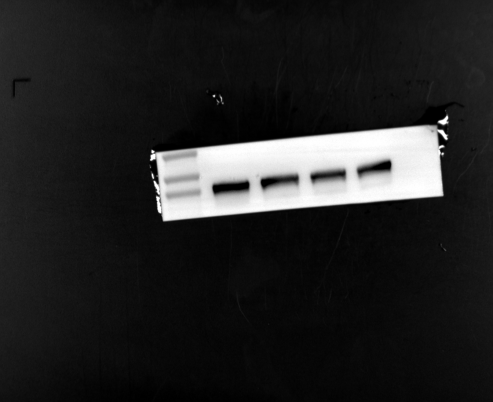


GAPDH

37kDa

p21

18kDa

Sucralose

Water

Sucralose

Water

Uterine


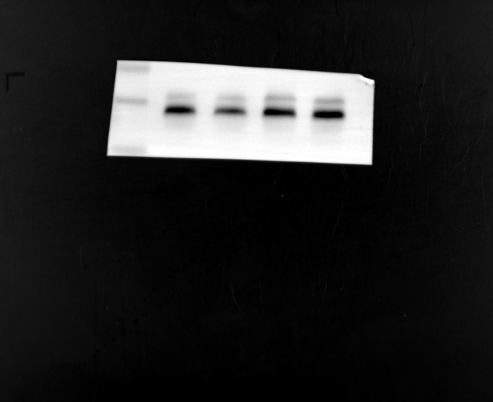

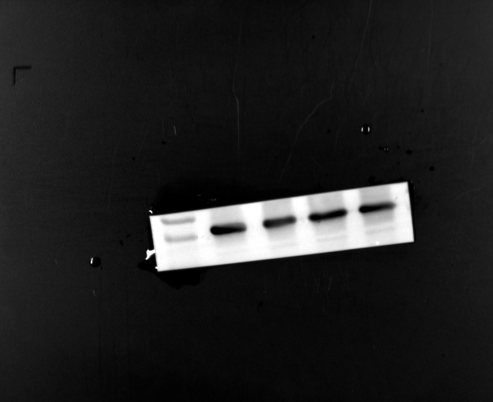


p21

18kDa

Sucralose

Water

Sucralose

Water

Kidney

GAPDH

37kDa


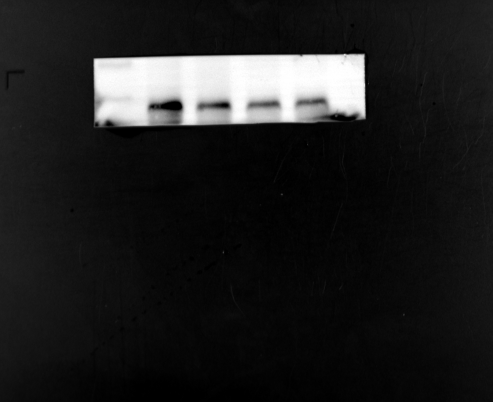

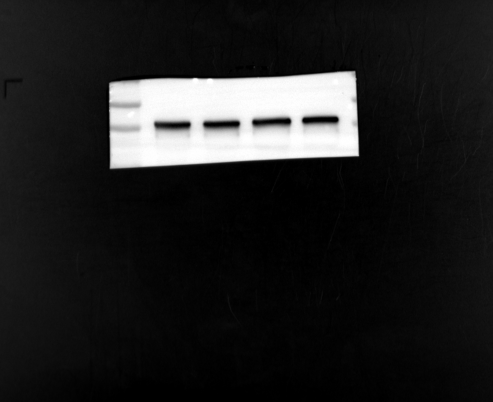


Sucralose

Water

GAPDH

37kDa

p21

18kDa

Sucralose

Water

Sucralose

Water

Liver


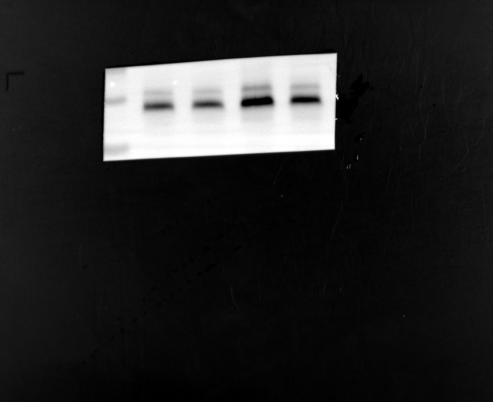

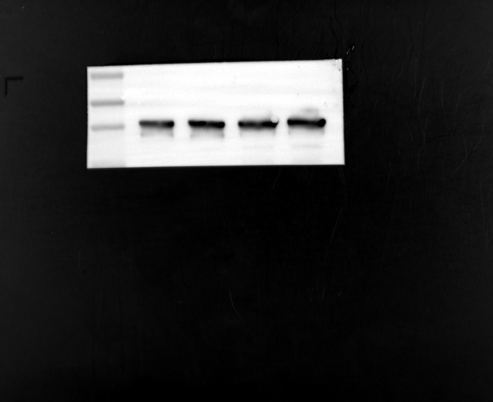


GAPDH

37kDa

p21

18kDa

Sucralose

Water

Heart

Figure 2 A

Water

Sucralose


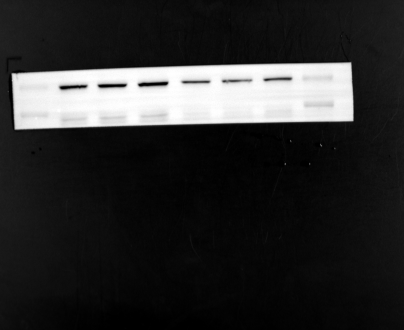

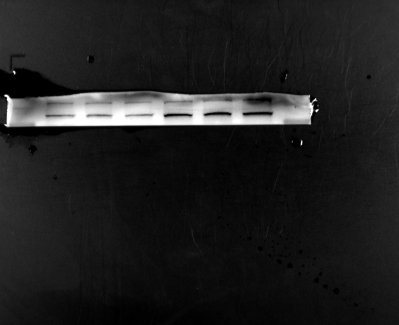

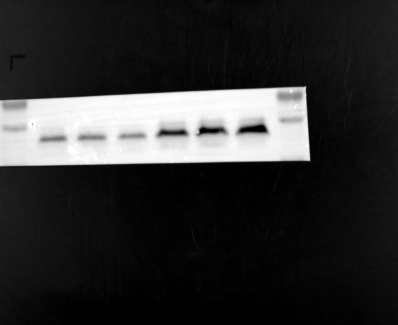


p21

18kDa

p53

53kDa

LaminB1

72kDa

Sucralose

Water

Water

Sucralose

Water

Sucralose


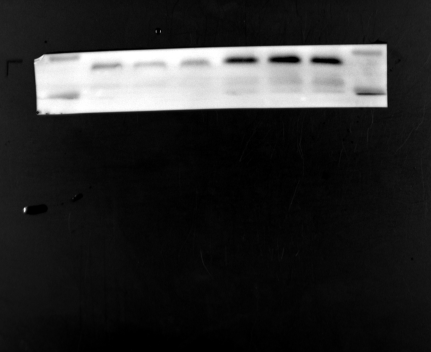

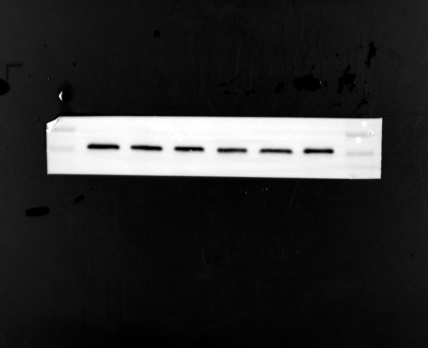


GAPDH

37kDa

p16

16kDa

Sucralose

Water

Figure 2C


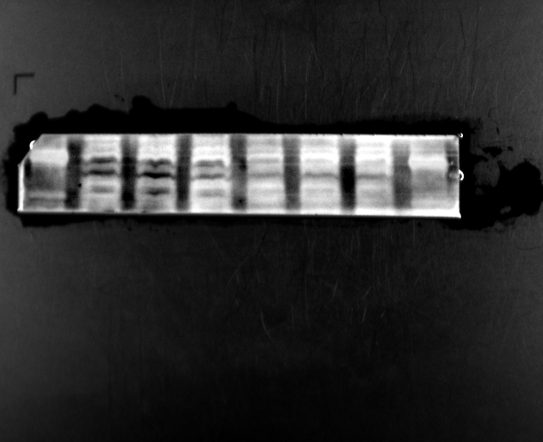

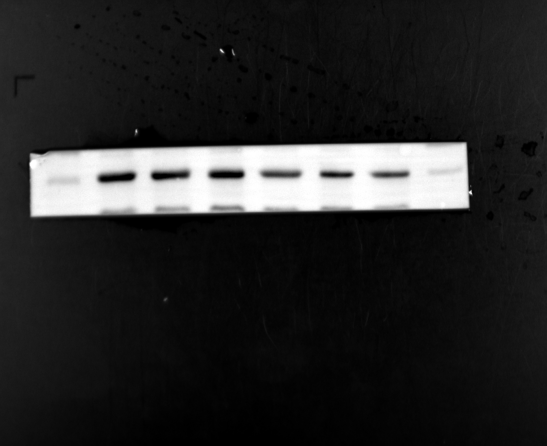


Sucralose

Sucralose

Water

Water

CYP11A150kDa

AMH

59kDa


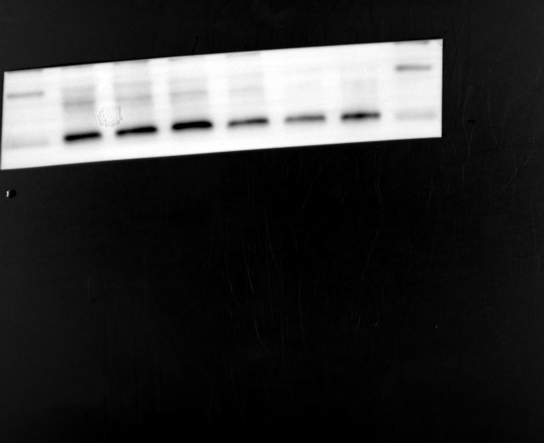

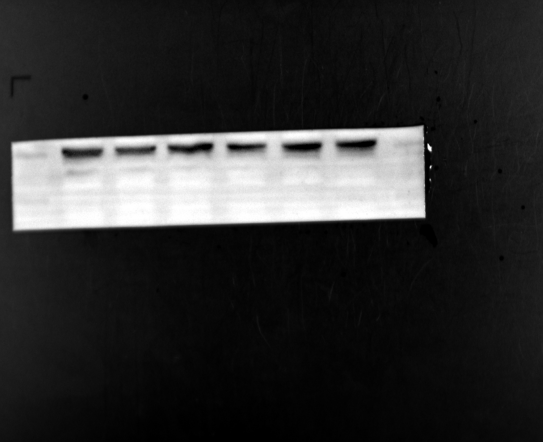


Sucralose

Sucralose

GAPDH

37kDa

StAR

32kDa

Water

Water

Figure 3F


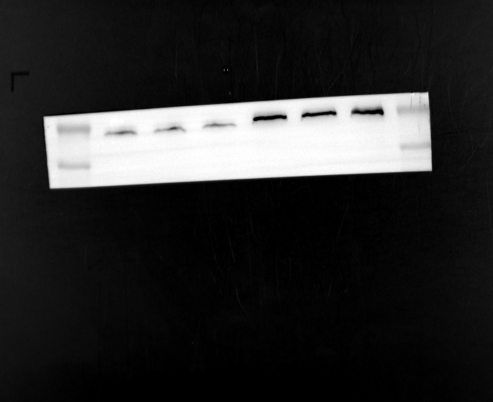

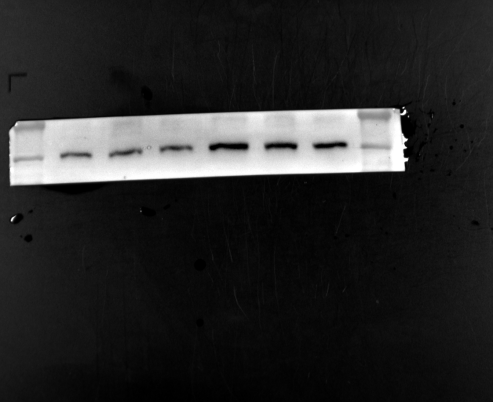


Sucralose

Sucralose

MyD88

35kDa

Water

Water

TLR4

90kDa


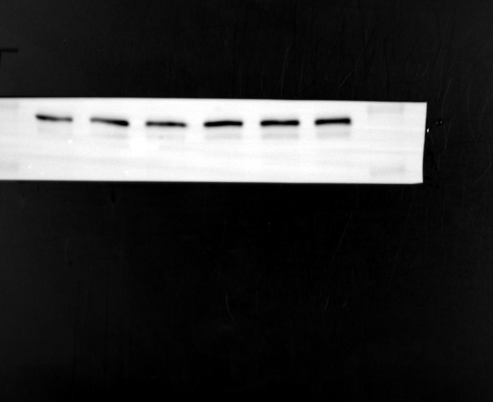

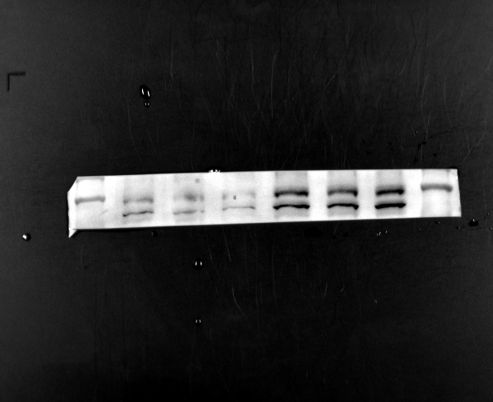


Sucralose

Sucralose

p65

65kDa

P-p65

65kDa

Water

Water


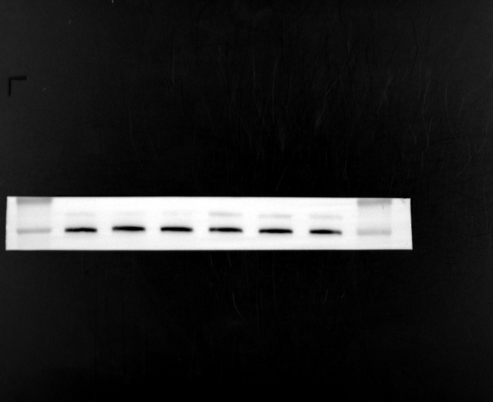


Sucralose

Actin

42kDa

Water

Figure 4F


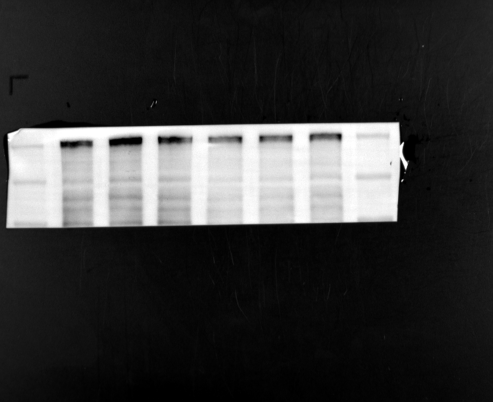

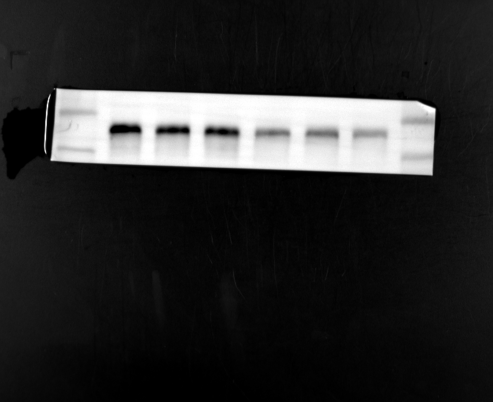


Sucralose

Sucralose

Water

Water

ZO-1

195kDa

Occludin

59kDa


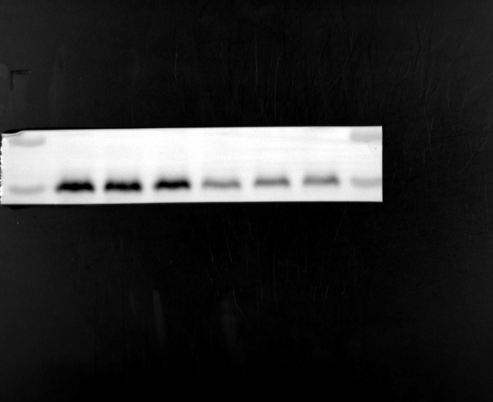

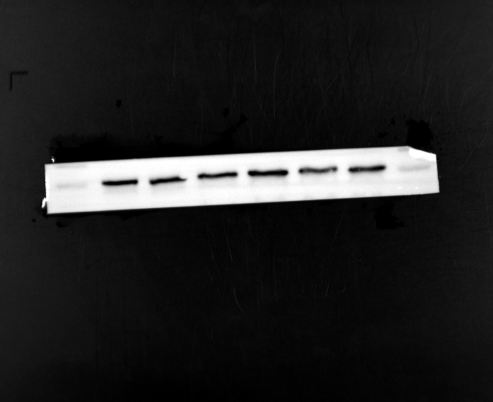


Sucralose

Sucralose

Water

Water

Claudin-1

23kDa

GAPDH

59kDa

Figure 5M


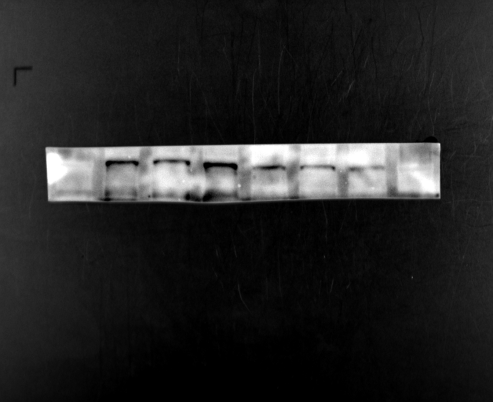

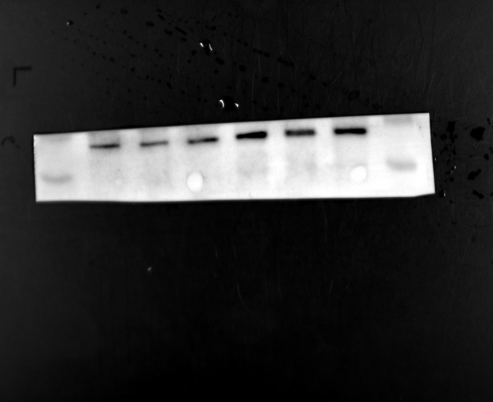


Sucralose-FMT

Water-FMT

Sucralose-FMT

Water-FMT

p16

16kDa

LaminB1

72kDa


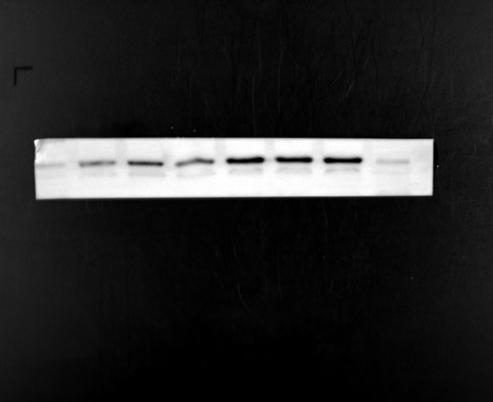

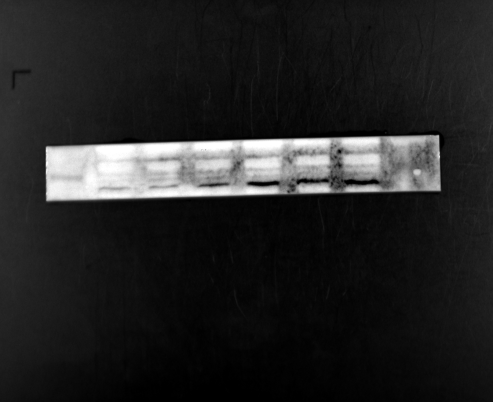


Sucralose-FMT

Water-FMT

Sucralose-FMT

Water-FMT

p53

53kDa

p21

18kDa


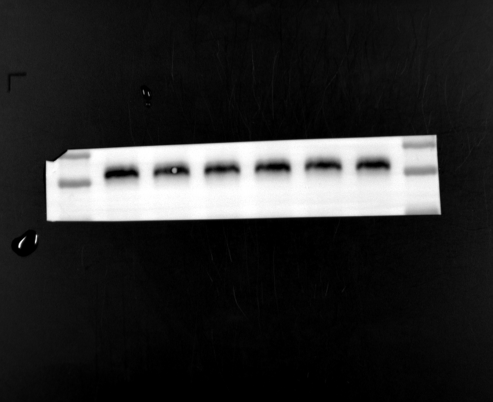


Sucralose-FMT

Water-FMT

GAPDH

37kDa

Figure 5O


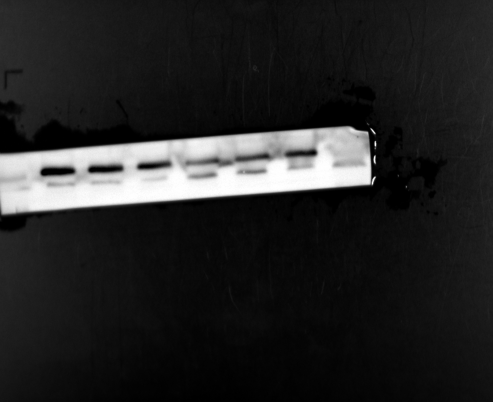

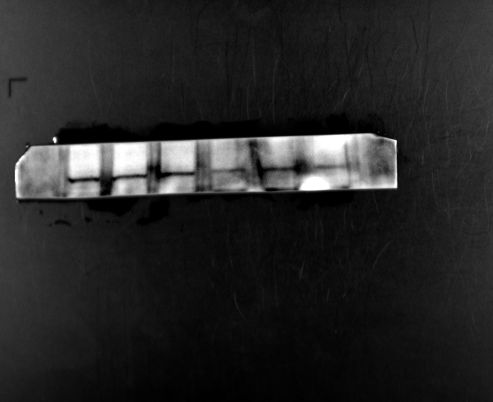


Water-FMT

Sucralose-FMT

Sucralose-FMT

Water-FMT

CYP11A150kDa

AMH

59kDa


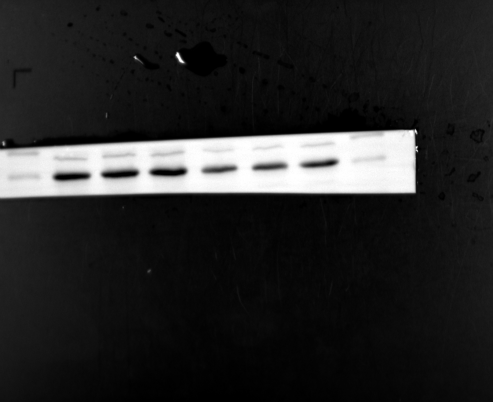

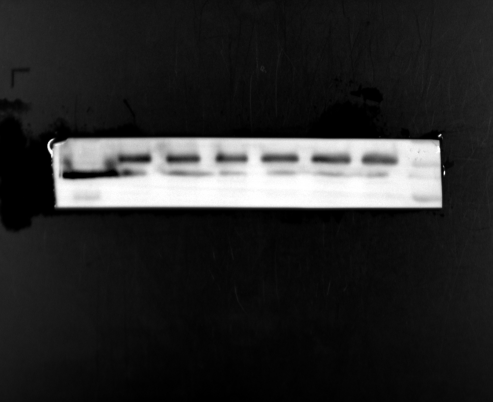


Water-FMT

Sucralose-FMT

Sucralose-FMT

Water-FMT

GAPDH

37kDa

StAR

32kDa

Figure 6B


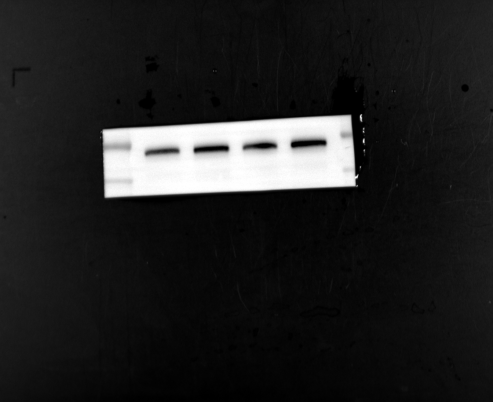

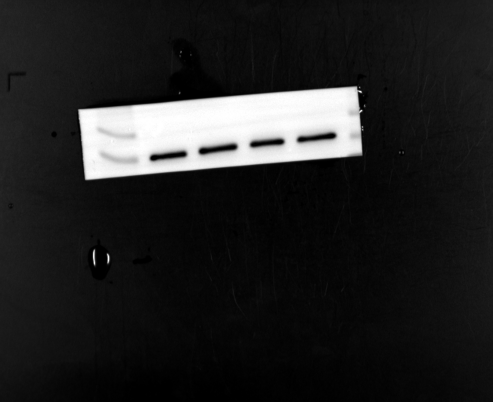


LPS

0 0.1 1.0 5.0

LPS

0 0.1 1.0 5.0

GAPDH

37kDa

p65

65kDa


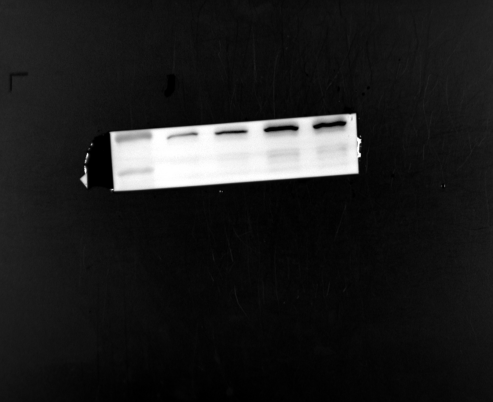


LPS

0 0.1 1.0 5.0

P-p65

65kDa

Figure 6E


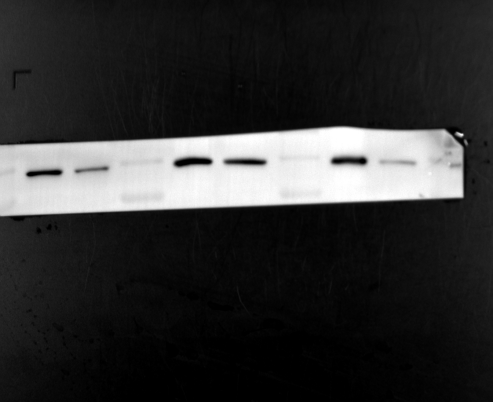

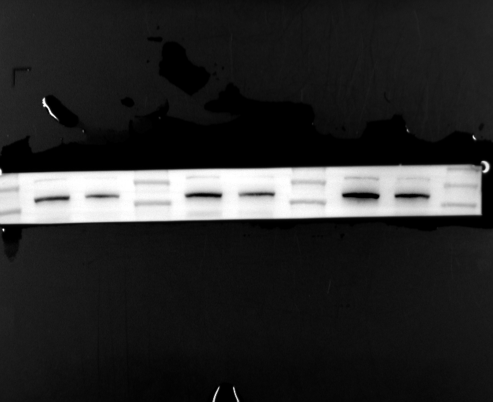


LPS

- +

LPS

- +

LPS

- +

AMH

59kDa

CYP11A150kDa


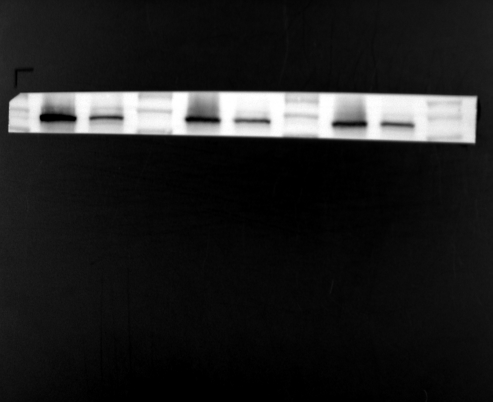

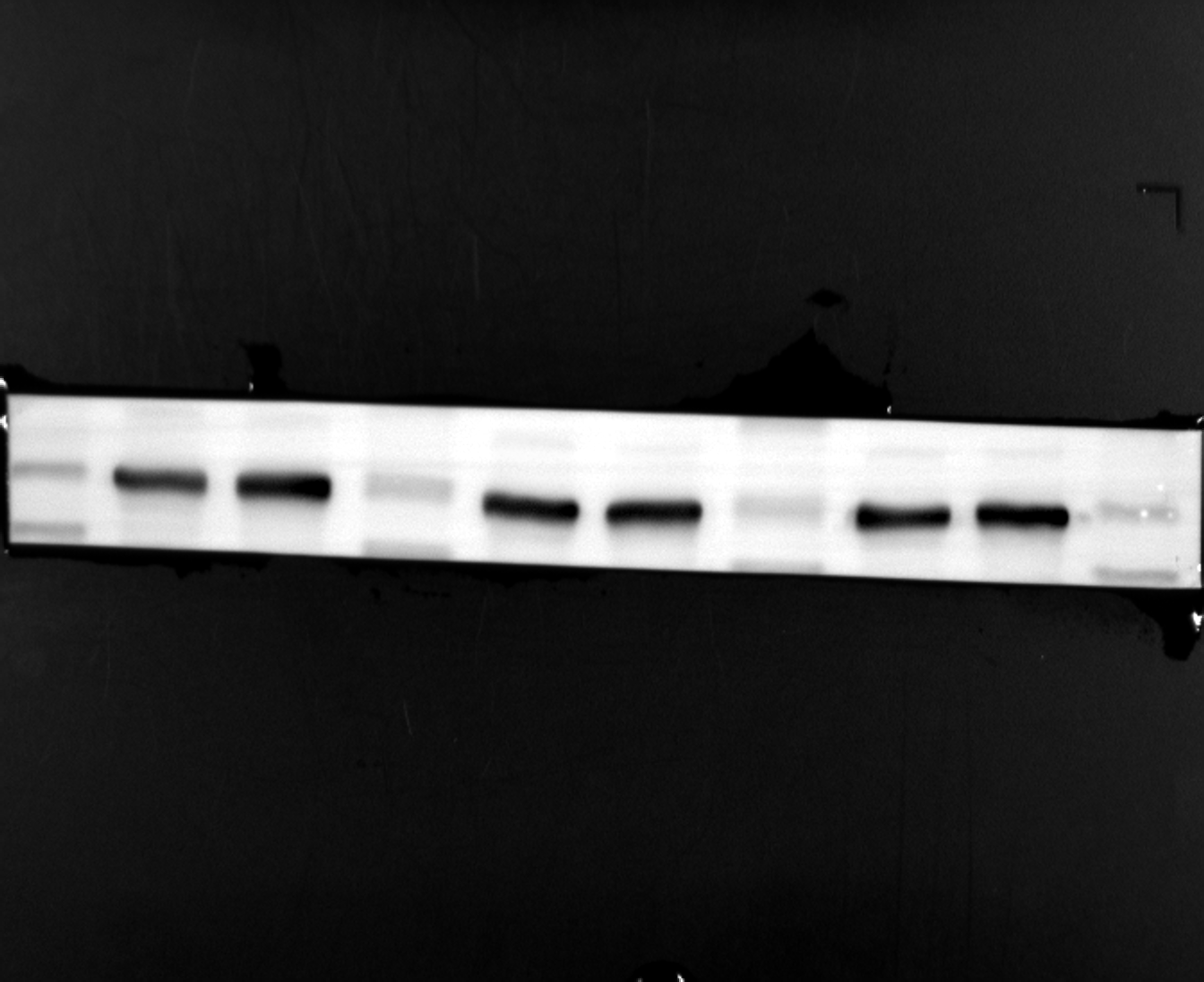


LPS

- +

GAPDH

37kDa

StAR

32kDa

Figure 6 I


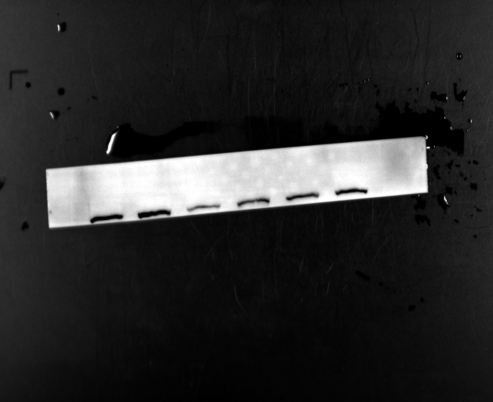

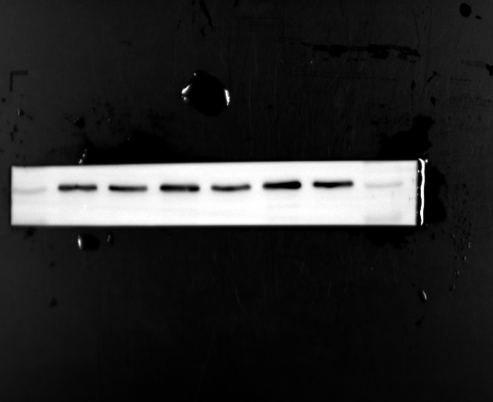


NC+sh-TLR4-2

LPS+sh-TLR4-2

NC+NC

NC+sh-TLR4-1

LPS+sh-TLR4-1

LPS+NC2

NC+sh-TLR4-2

LPS+sh-TLR4-2

NC+NC

NC+sh-TLR4-1

LPS+sh-TLR4-1

LPS+NC2

LPS+sh-TLR4-2

NC+NC

NC+sh-TLR4-1

LPS+sh-TLR4-1

LPS+NC2

NC+sh-TLR4-2

NC+sh-TLR4-2

LPS+sh-TLR4-2

NC+NC

NC+sh-TLR4-1

LPS+sh-TLR4-1

LPS+NC2

TLR4

90kDa

p65

65kDa


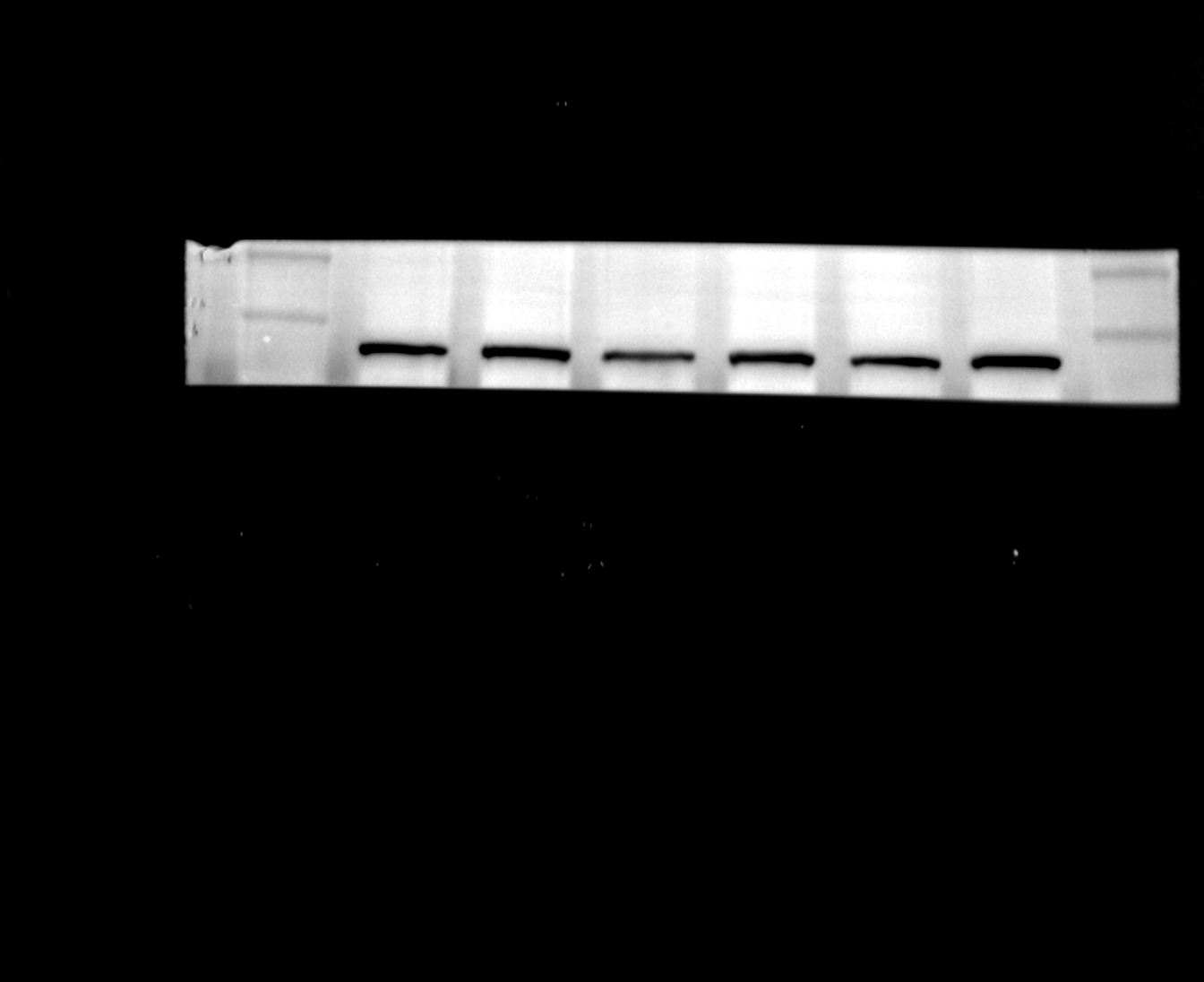

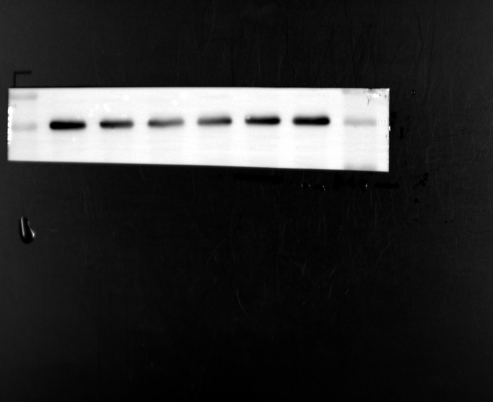


P-p65

65kDa

GAPDH

37kDa

NC+NC

LPS+NC

NC+TAK-242

LPS+TAK-242

LPS+TAK-242

Figure 6 J

NC+TAK-242

NC+NC

LPS+NC


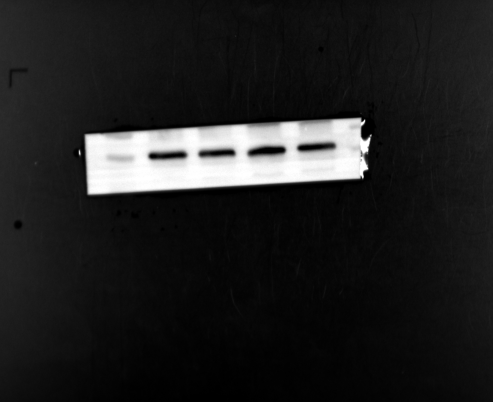

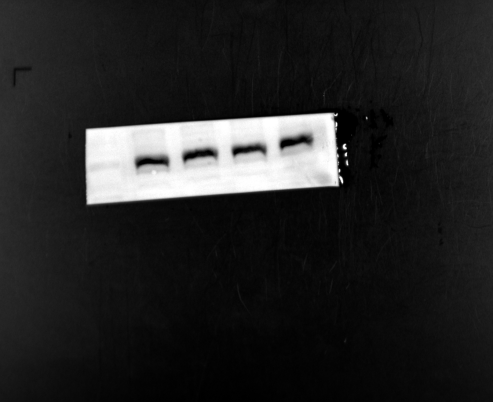


LPS+TAK-242

NC+TAK-242

NC+NC

LPS+NC

LPS+TAK-242

NC+TAK-242

NC+NC

LPS+NC

p65

65kDa

GAPDH

37kDa


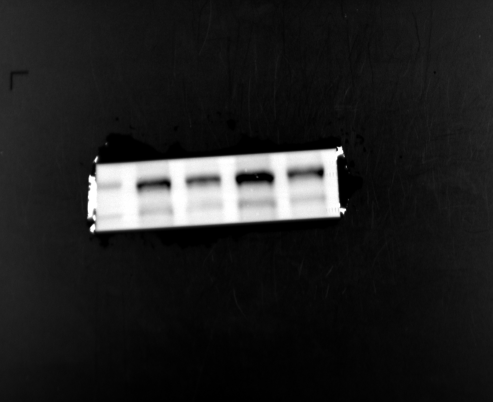

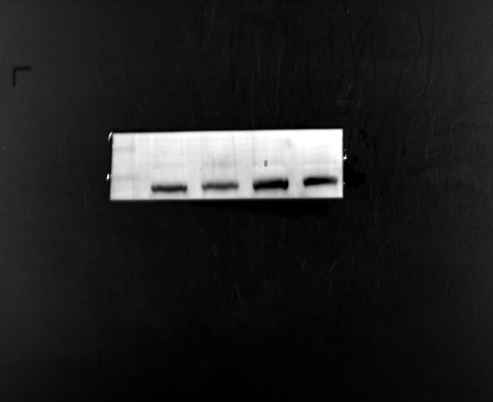


P-p65

65kDa

TLR4

90kDa

Figure 6 K

NC+NC

LPS+sh-TLR4

LPS+NC

NC+NC

LPS+sh-TLR4

LPS+NC

LPS+NC

NC+NC

LPS+sh-TLR4


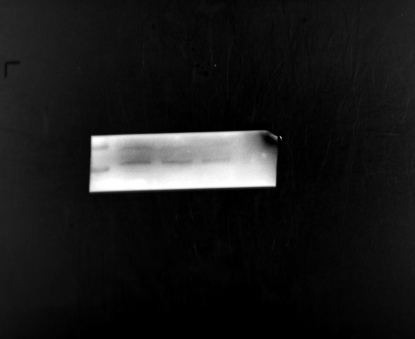

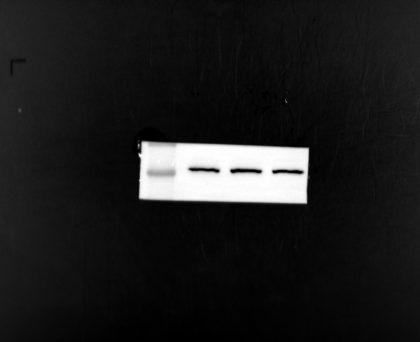

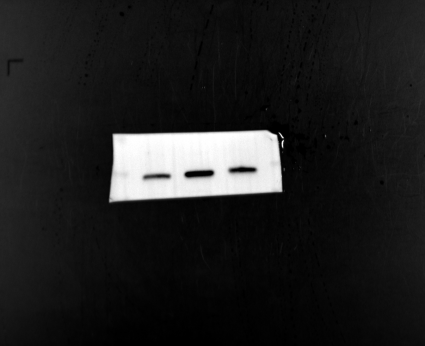


NC+NC

LPS+sh-TLR4

LPS+NC

NC+NC

LPS+sh-TLR4

LPS+NC

NC+NC

LPS+sh-TLR4

LPS+NC

LaminB1

72kDa

p65

65kDa

GAPDH

37kDa


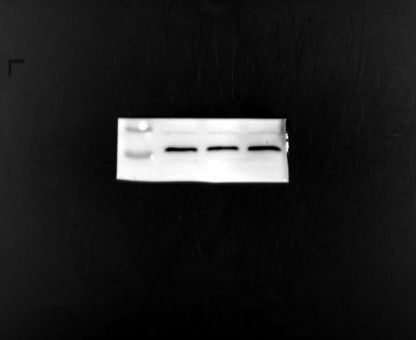

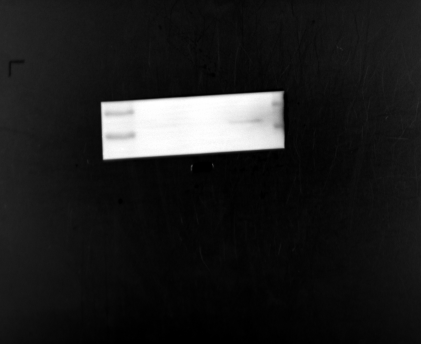

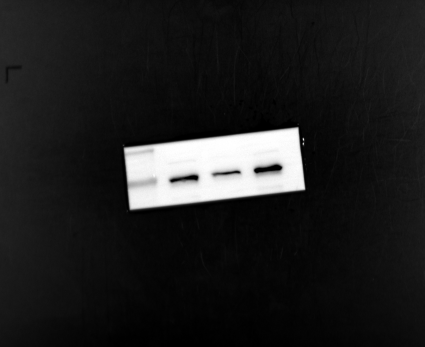


p65

65kDa

LaminB1

72kDa

GAPDH

37kDa

Sucralose+BME-Low

Sucralose+BME-High

Sucralose+BME-High

Sucralose+BME-Low

Sucralose+NC

NC+NC

Sucralose+NC

NC+NC

Figure 7 J


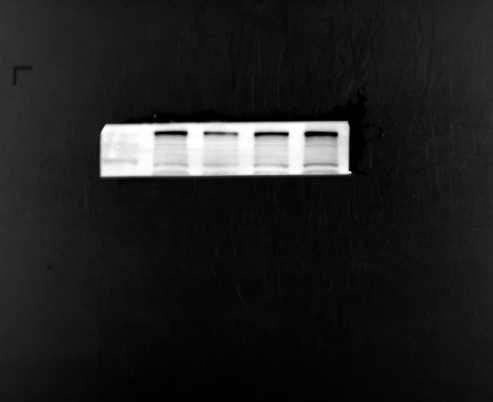

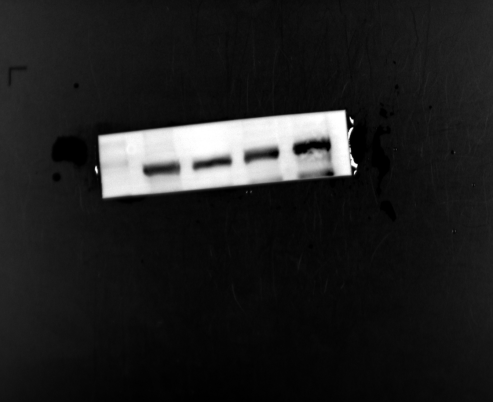


Sucralose+BME-High

Sucralose+BME-Low

NC+NC

Sucralose+BME-High

Sucralose+BME-Low

Sucralose+NC

NC+NC

Occludin

59kDa

ZO-1

195kDa


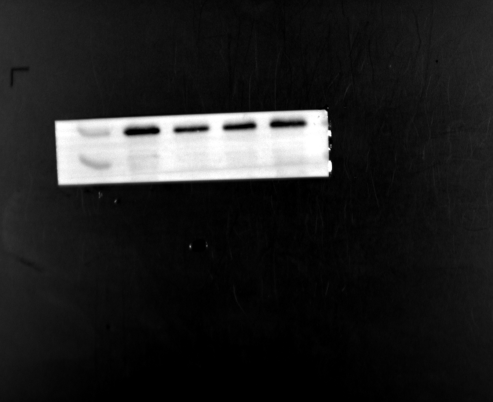

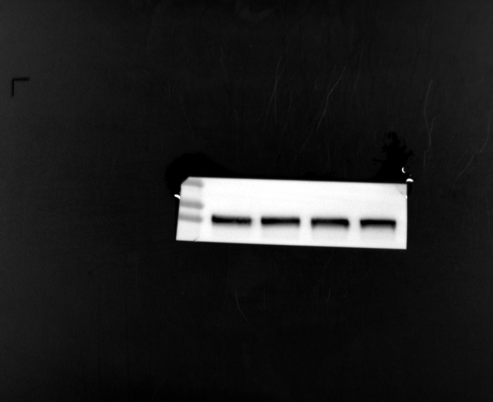


Sucralose+NC

GAPDH

59kDa

Claudin-1

23kDa

Figure 8A


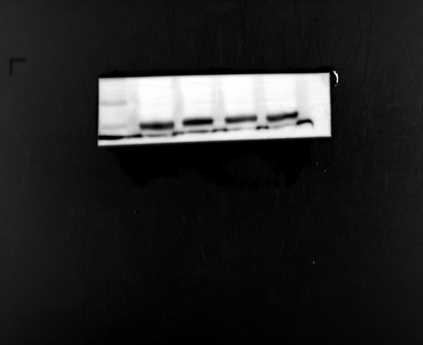

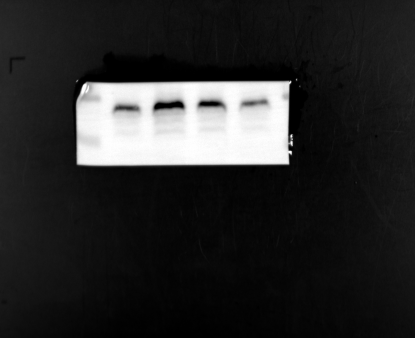

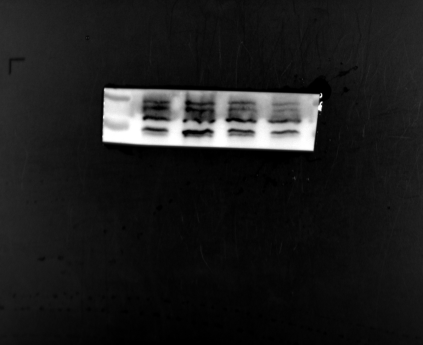


Sucralose+BME-High

Sucralose+BME-Low

Sucralose+NC

NC+NC

Sucralose+BME-High

Sucralose+BME-Low

Sucralose+NC

NC+NC

Sucralose+BME-High

Sucralose+BME-Low

Sucralose+NC

NC+NC

GAPDH

37kDa

p16

16kDa

p21

18kDa

Figure 8 D

Sucralose+BME-High

Sucralose+BME-Low

Sucralose+BME-High

Sucralose+BME-Low

Sucralose+NC

NC+NC

Sucralose+NC

NC+NC


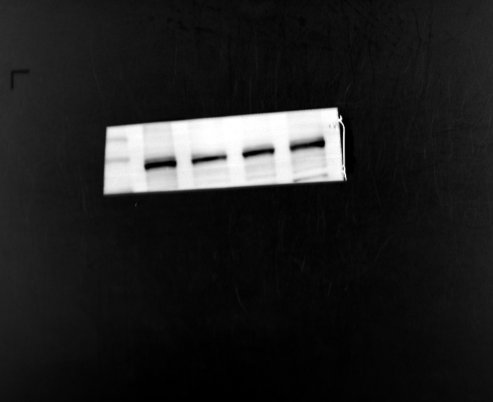

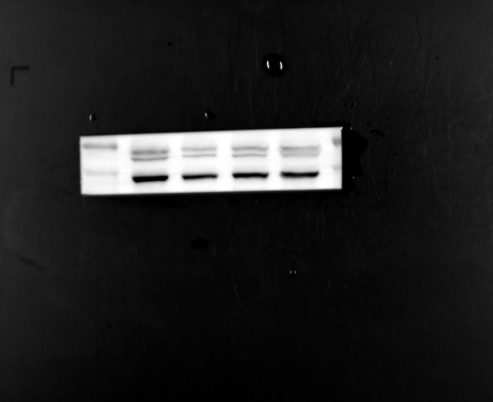


Sucralose+BME-High

Sucralose+BME-Low

Sucralose+BME-High

Sucralose+BME-Low

CYP11A150kDa

AMH

59kDa


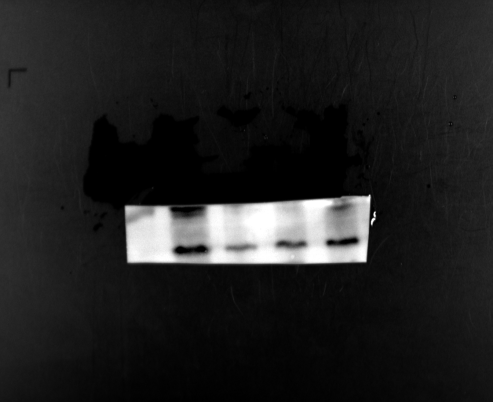

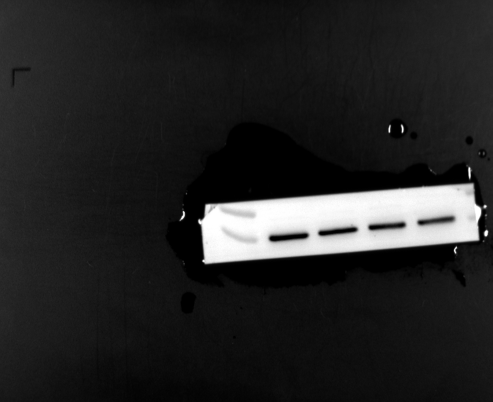


Sucralose+NC

NC+NC

Sucralose+NC

NC+NC

GAPDH

37kDa

StAR

32kDa

Sucralose+BME-High

Sucralose+BME-Low

Sucralose+BME-High

Sucralose+BME-Low

Sucralose+NC

NC+NC

Figure 8 I

Sucralose+NC

NC+NC


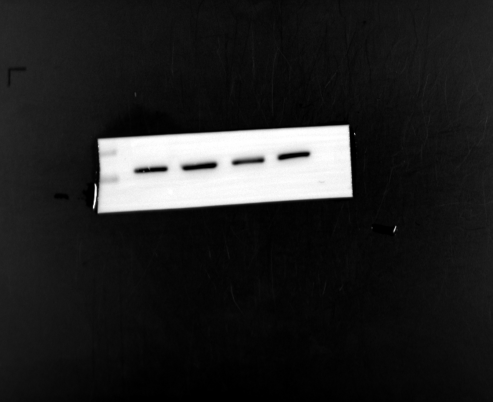

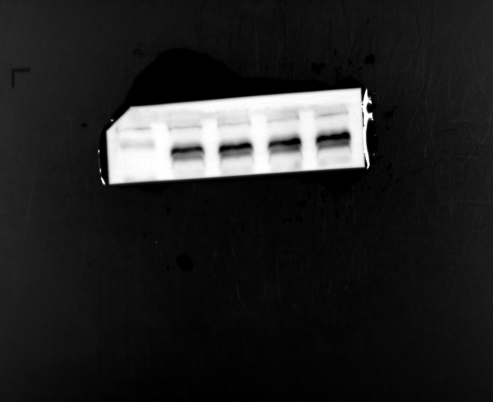


GAPDH

37kDa

p65

65kDa


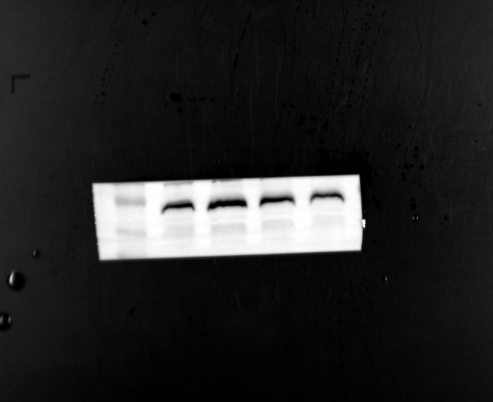

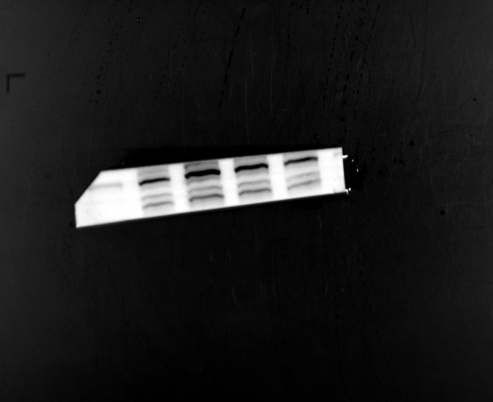


TLR4

90kDa

P-p65

65kDa

Sucralose+BME-Low

Sucralose+NC

NC+NC

Sucralose+BME-High

Sucralose+BME-High

Sucralose+BME-Low

Sucralose+NC

NC+NC

Figure S1B

Figure 1 B


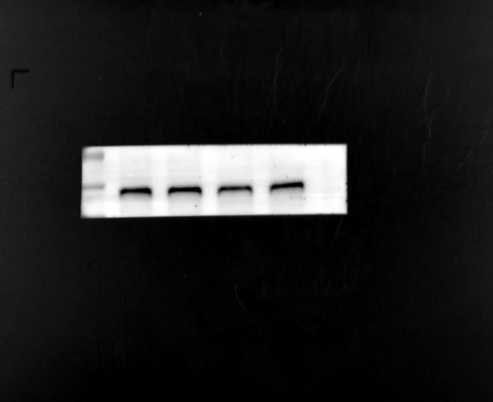

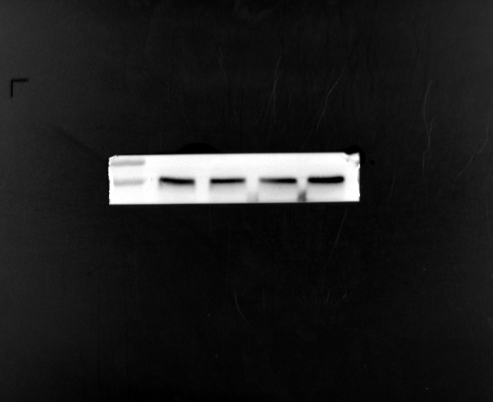


GAPDH

37kDa

p21

18kDa

Sucralose

Water

Sucralose

Water

Ovary


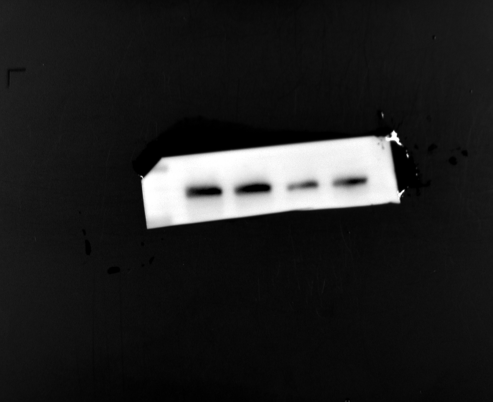

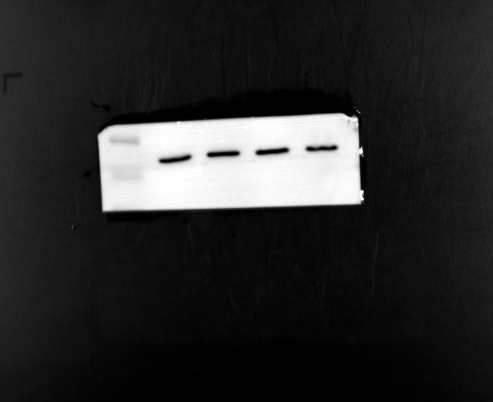


Water

Sucralose

Water

Sucralose

GAPDH

37kDa

p21

18kDa

spleen


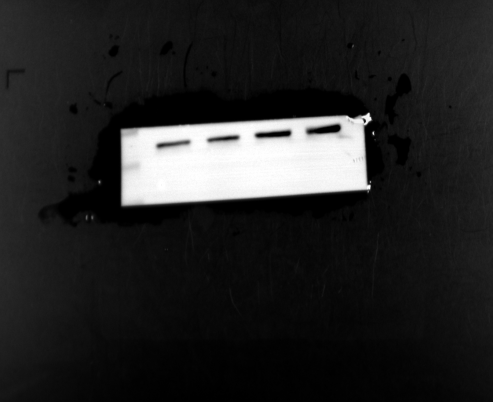

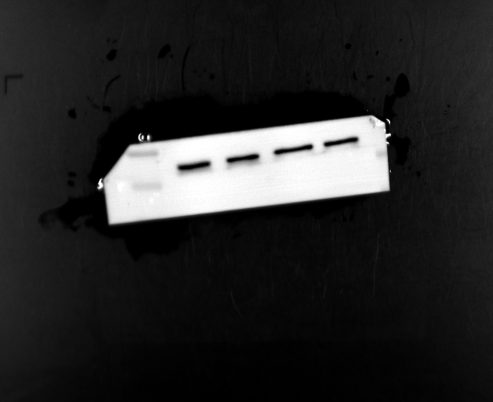


p21

18kDa

GAPDH

37kDa

Sucralose

Water

Sucralose

Water

Uterine


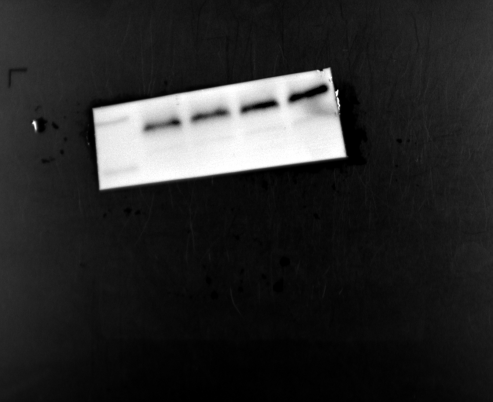

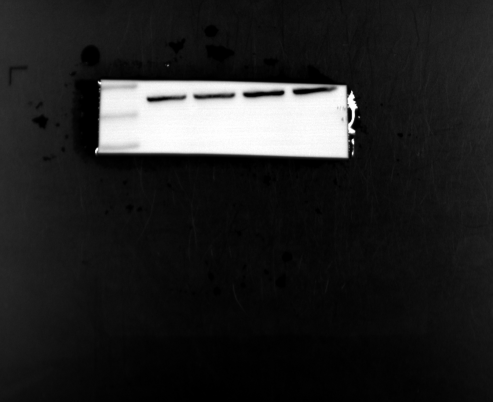


GAPDH

37kDa

Water

Sucralose

p21

18kDa

Sucralose

Water

Kidney


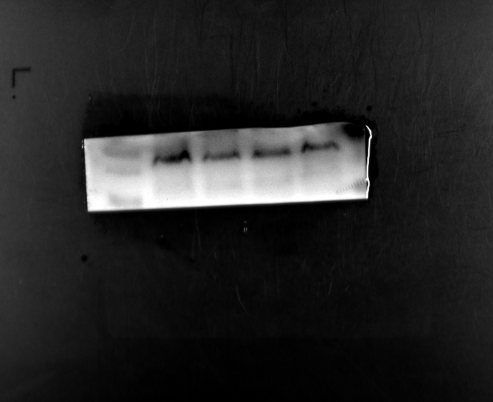

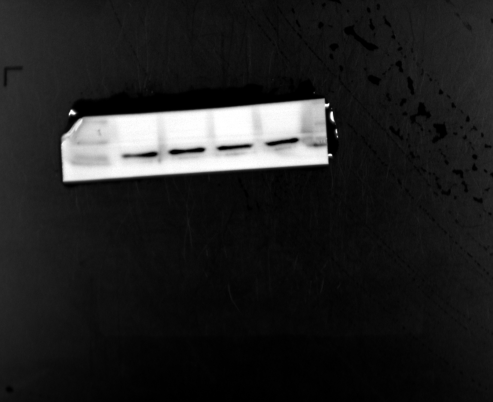


Sucralose

Water

Sucralose

Water

p21

18kDa

GAPDH

37kDa

Liver


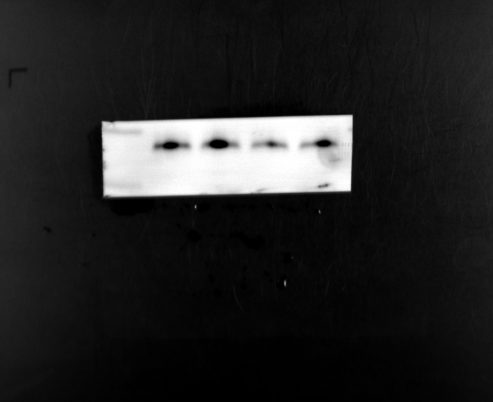

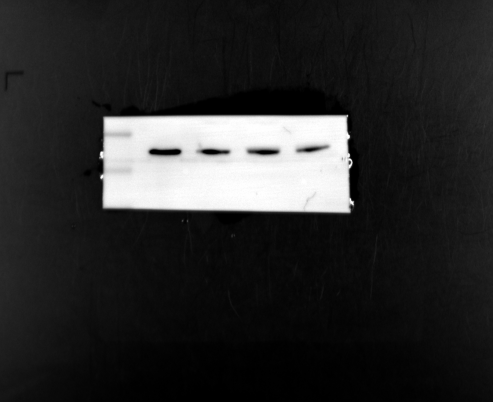


p21

18kDa

GAPDH

37kDa

Sucralose

Water

Sucralose

Water

Heart

FigureS3A


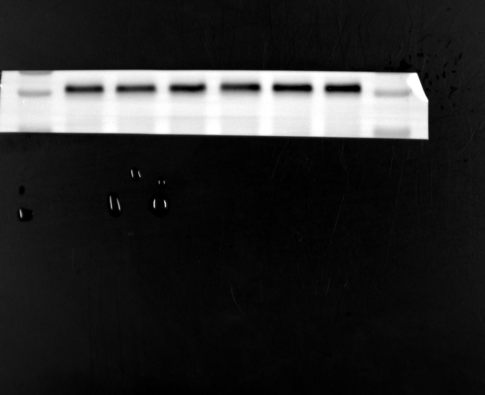

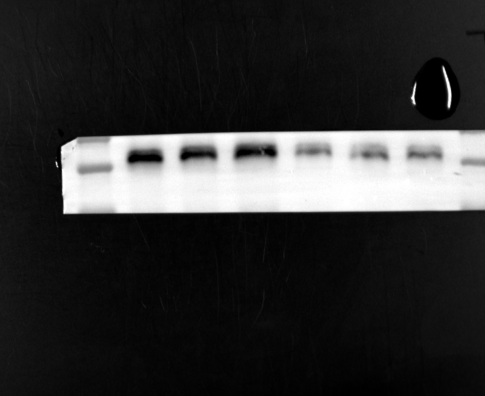


p-IKBα

37kDa

IKBα

37kDa

Water

Sucralose

Water

Sucralose


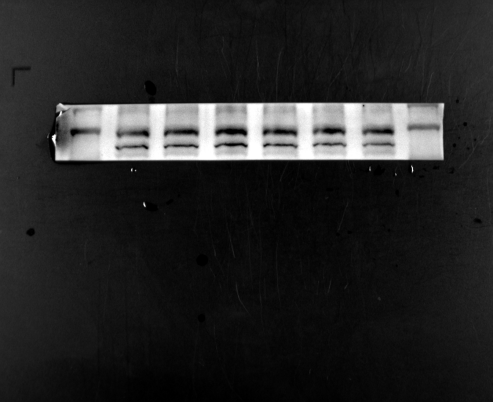

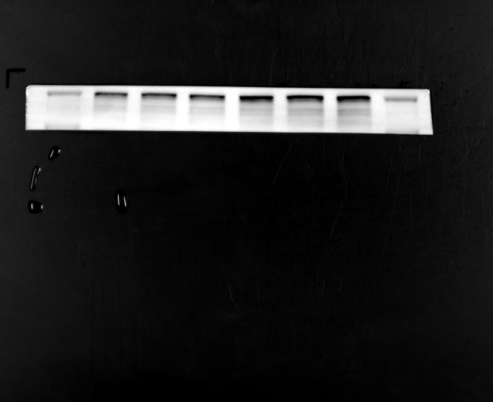


IKKα

85kDa

Water

Sucralose

p-IKKα

85kDa

Water

Sucralose

Water


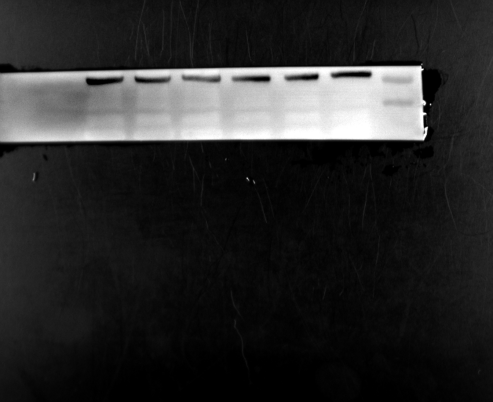


TUBULIN55kDa

Sucralose
